# Protocol: What works to increase the use of evidence for policy decision‐making: A systematic review

**DOI:** 10.1002/cl2.1435

**Published:** 2024-11-22

**Authors:** Promise Nduku, John Ategeka, Andile Madonsela, Tanya Mdlalose, Jennifer Stevenson, Shannon Shisler, Suvarna Pande, Laurenz Mahlanza‐Langer

**Affiliations:** ^1^ Pan‐African Collective Evidence (PACE) Johannesburg South Africa; ^2^ International Initiative for Impact Evaluation 3ie

**Keywords:** evidence‐informed decision‐making, knowledge translation, meta‐analysis, policymaking, research use, systematic review

## Abstract

This is the protocol for a Campbell systematic review. The objectives are as follows: Our aim is to collect, assess, and synthesise all the available empirical evidence on what works to support evidence‐informed decision‐making by policymakers. In doing so, we will aim to answer the following research questions: What are the impacts of interventions to support evidence‐informed decision‐making by policymakers? What are the factors which have influenced the impact of these interventions, and their design and implementation in low‐ and middle‐income countries? In answering these questions, our goal is to estimate the overall impact and relative effectiveness of different interventions, identify factors or configurations of factors that support or hinder their effectiveness in low‐ and middle‐income countries and to identify gaps and areas for future primary research.

## 1 BACKGROUND

### 1.1 The issue

Effective and equitable public policies and programmes present a major pathway for socio‐economic development. The systematic use of data and evidence during decision‐making is a cornerstone in the design and implementation of such policies and programmes. Such evidence‐informed decision‐making (EIDM) can improve policies and programmes in at least three ways. First, from an economic perspective, the use of evidence allows decision‐makers to zoom in on the most impactful and cost‐effective policies maximising the gains of the investment of scarce public resources. Second, from a political perspective, using evidence and data transparently during decision‐making can enhance accountability and citizen's trust in policymaking and proposed policies and programmes. Third, from an equity perspective, data and evidence can serve as a proxy for groups and viewpoints traditionally excluded from decision‐making contexts. To advance policies and programmes to tackle inequities, data and evidence is required to substantiate the extent of these and how they can best be addressed.

However, data and evidence are by far not the only input for policy decision‐making and other factors such as politics, contexts, ideologies, budget considerations, and so forth, play an equally important role. This has been acknowledged since the inception of the evidence movement in the healthcare sector with the first models for evidence‐based medicine explicitly defining evidence as one input for decision‐making in a practice setting (with the other two being clinical expertise and patient values) (Sackett & Rosenberg, 1995). Decision‐makers are usually supported by a range of policy and research professionals, both within government as well as in trusted organisations, who give advice, and prepare and collate information and evidence on their behalf. These actors, too, therefore play crucial roles in the EIDM process.

Policy‐focused research has often relied on the assumption that generating more high‐quality evidence will increase uptake at different stages of the policy cycle. Oliver et al.'s (2014) systematic review of the barriers and facilitators to the use of evidence in policymaking indicated a number of reasons why this is not always the case. Across sectors, the gulf between researchers and policymakers, unclear, irrelevant, low‐quality evidence, and lack of timelines or opportunities were identified as significant barriers to implementing EIDM.

In addition, much of the previous research on the use of evidence in public policymaking engaged in a limited manner with the political and institutional nature of decision‐making (Parkhurst, 2017). This is a limitation that the Strengthening the Use of Evidence for Development Impact (SEDI) programme, funded by the UK Foreign, Commonwealth and Development Office (FCDO), aimed to rectify. Working in Uganda, Pakistan and Ghana, the project began by undertaking a political economy analysis of evidence use in each country to ensure that the programme's subsequent approach to the intervention was informed by an in‐depth understanding of the context (Shaxson et al., 2021). Analysis of a country's current context relating to the use of evidence in decision‐making and an assessment of the evidence ecosystem is also recommended by the WHO's guidelines on supporting the routine use of evidence during the policy‐making process (WHO, 2023).

Oliver et al.'s (2022) systematic review identified a significant expansion of research‐policy engagement initiatives to encourage greater use of evidence in decision‐making, finding 1923 initiatives being undertaken by 513 organisations globally. This included initiatives to build decisionmaker skills around evidence use, promoting engagement through incentives and rewards and building professional partnerships. However, they found that a significant proportion of this dynamic activity is going unevaluated.

Despite this finding, there is an increasing number of robust counterfactual evaluations that test the impact of strategies to encourage EIDM using experimental and quasi‐experimental methods. Hjort et al. (2021) conducted an experiment with 1818 municipality mayors in Brazil, where half the mayors were invited to attend a research‐information session on the effectiveness of taxpayer reminder letters as demonstrated by consistent randomised controlled trial (RCT) evidence. Fifteen to 24 months later, they found that simple approach providing access to research evidence increased the probability that the tax policy gets implemented by 10 percentage points. In the United States, Crowley et al. (2021) evaluated the impact of a formal outreach model between federal lawmakers working on child and family policy issues and researchers to encourage congressional use of research evidence. They observed positive impacts on a range of evidence‐use‐related outcomes, including research use observed in legislation brought in by the treatment group of congressional offices, as well as the greater value of research for understanding policy. There is also a valuable body of other types of primary research around initiatives to strengthen EIDM, including cross‐country efforts such as Vogel and Punton (2018) and Lester et al. (2020) that speak to the question of who, when and for whom these initiatives are effective.

### 1.2 Description of the conceptual framework for the review

This systematic review is concerned with interventions able to enhance and support the use of evidence in policy decision‐making. In the absence of an agreed‐on over‐arching theory of how EIDM occurs, we will apply and refine a conceptual framework developed by Langer and colleagues for the Art and Science of Using Evidence project (Langer et al., 2016; Nduku et al., 2024). This framework encompasses interventions targeting evidence use in decision‐making, categorised according to six identified mechanisms of change, which are the processes through which EIDM can be achieved. The primary outcome of interest is the behaviour of using evidence, which can be further broken down into the intermediary components of capability, opportunity, and motivation (COM) to use evidence. We acknowledge that EIDM interventions can target behaviour change at various levels, including individuals and organisations. These four elements—evidence use interventions, mechanisms of change, behavioural outcomes, and levels of intervention—serve as the conceptual device for organising and assessing the evidence base.

We will categorise evidence use interventions based on the underlying mechanisms of change. Langer et al. (2016) identified six such mechanisms from previous studies (e.g., Gough et al., 2011; Nutley et al., 2007), barriers and facilitators research on decision‐makers' use of evidence (e.g., Oliver et al., 2014), and existing empirical intervention frameworks (e.g., Moore et al., 2011). Interventions aimed at increasing EIDM are assumed to operate through individual mechanisms or a combination of mechanisms. Table 1 below outlines these six mechanisms. These interventions could include programmes, strategies, actions, or practices that actively modify the current decision‐making status quo to make it more receptive to evidence use. Examples of EIDM interventions include professional development activities to enhance policymakers' awareness and capacity to use evidence in policy development, convening communities of practice, and implementing rapid response services that provide quick syntheses of research findings in response to policymakers' requests.

**Table 1:** Conceptual framework to structure EIDM interventions according to mechanisms of change.

Mechanism	Description	Example of linked activity
Awareness (M1)	Building awareness for, and positive attitudes towards, evidence‐informed decision‐making (EIDM). This mechanism emphasises the importance of decision‐makers' valuing the concept of EIDM.	– Social marketing of the norm to use evidence (e.g., Sense About Science) – Awareness raising campaigns (e.g., March for Science)
Agree (M2)	Building mutual understanding and agreement on policy‐relevant questions and the kind of evidence needed to answer them. This mechanism emphasises the importance of building mutual understanding and agreement on policy questions and what constitutes fit‐for‐purpose evidence.	– Co‐production approaches – Delphi panels – Inter‐professional education
Access (M3)	Providing communication of, and access to, evidence. This mechanism emphasises the importance of decision‐makers receiving effective communication of evidence and convenient access to evidence.	– Knowledge repositories – Communication campaigns and strategies – Policy briefs
Interact (M4)	Interaction between decision‐makers and researchers.^a^ This mechanism emphasises the importance of decision‐makers interacting with researchers to build trusted relationships, collaborate, and gain exposure to a different type of social influence.	– Networks and communities of practice – Events and conferences (e.g., science cafés) – Knowledge brokers
Skills (M5)	Supporting decision‐makers to develop skills in accessing and making sense of evidence. This mechanism emphasises the importance of decision‐makers having the necessary skills to locate, appraise, synthesise evidence, and integrate it with other information and political needs, etc.	– Capacity‐building (e.g., workshops and formal training courses) – Mentoring programmes – Adult learning – Online learning
Structure & Process (M6)	Influencing decision‐making structures and processes. This mechanism emphasises the importance of decision‐makers' psychological, social, and environmental structures and processes (e.g., mental models, professional norms, habits, organisational and institutional rules) in providing means and barriers to action.	– Secondments – Organisational supports (e.g., embedded knowledge brokers) – Rapid Response Services – Institutionalisation (e.g., National Evaluation Systems) – Evidence checklists

^a^ Use of the term researcher denotes anyone conducting research and is not confined to appointed individuals in official research positions.

*Source*: Langer et al. (2016).

We will not include evaluations of interventions that focus only on enhancing the supply of research, such as financial incentives to produce better‐quality or more research. While supply‐side interventions can be an important tool to enhance EIDM, the focus of this systematic review is on the more direct use of evidence by policy decision‐makers. Interventions such as research co‐production, engagement and rapid response services are of relevance to this review however as they target decision‐makers’ demand for evidence.

### 1.3 How the intervention might work

**Figure 1:**
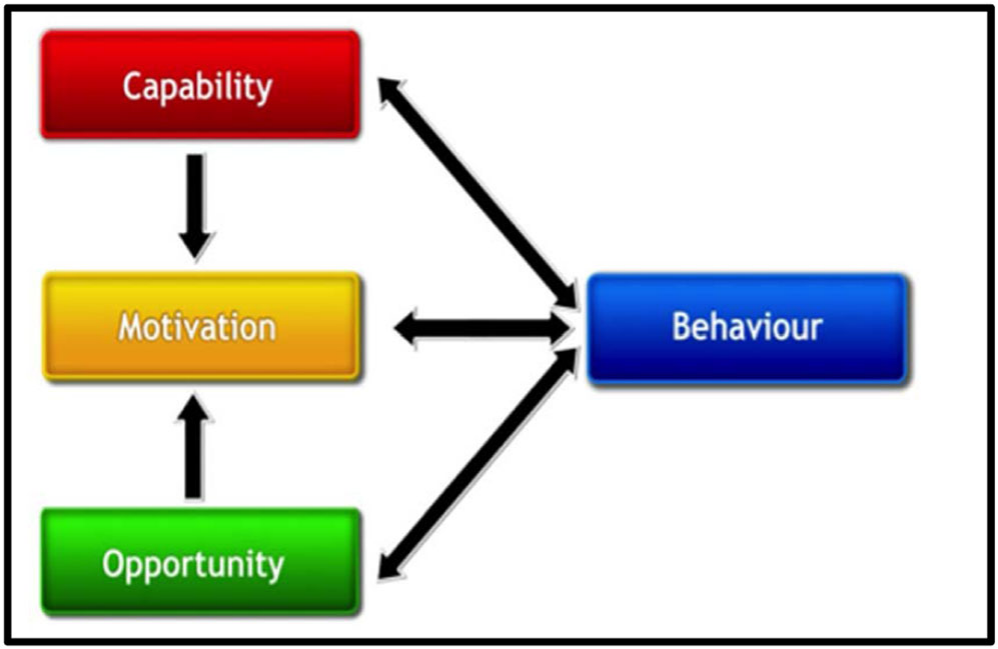
Components of behaviour change. *Source*: Michie et al. (2011).

Increasing the use of evidence by decision‐makers relies on behaviour change. Specifically, this involves decision‐makers using evidence to influence policy debates, policy choices, and policy implementation. Michie et al. (2011) developed a method to characterise interventions and link them to an analysis of the targeted behaviour. In this ‘behaviour system’, three essential conditions—COM—interact to generate behaviour, which in turn influences these components. These conditions influencing behaviour change therefore constitute the intermediary outcomes in addition to the main outcome behaviour of EIDM. Any given intervention might alter one or more components in this ‘behaviour system’ (see Figure 1). Our systematic review has adopted Michie's definitions of capability, motivation, and opportunity, which we define as the capability, motivation, and opportunity to use evidence.^1^

Behaviour change can occur at both the organisational and individual levels. For this systematic review, behaviour is categorised into four levels consisting of:

1. Individual behaviour
2. Team‐level behaviour
3. Organisational behaviour (e.g., a government ministry, an individual NGO)
4. Institutional behaviour (e.g., government‐wide, system‐specific)

**Figure 2:**
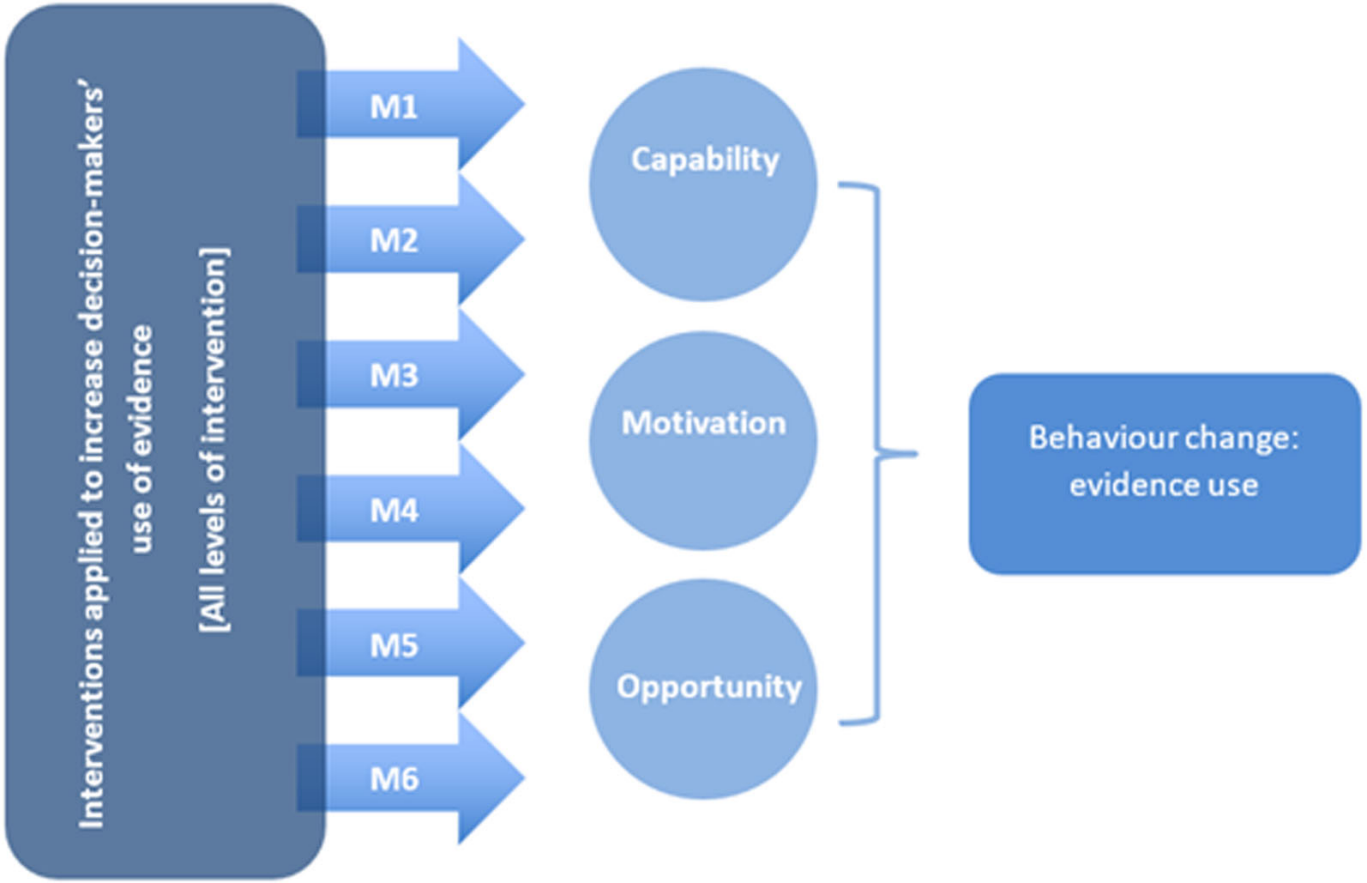
EIDM intervention logic model based on Science of Using Science conceptual framework. *Source*: Langer et al. (2016).

As noted above, there is no theoretical consensus explaining how interventions can effectively influence decision‐makers' use of evidence. Langer et al. (2016) therefore integrated the individual components of this conceptual framework to create a simple logic model that outlines how evidence use interventions are assumed to affect decision‐makers' consideration of evidence (Figure 2). This model illustrates how interventions may influence evidence use through a single mechanism or a combination of multiple mechanisms. By applying these mechanisms, interventions can affect one or more components of behaviour change, namely capability, opportunity, and/or motivation to use evidence. These COM components then facilitate the ultimate outcome of evidence use. Thus, a COM component can be understood as an intermediate outcome on the causal pathway to the final outcome. COMs can function either independently or in combination.

Using this logic model will allow us to categorise the interventions according to the applied intervention mechanisms (M1–M6, outlined in Table 1). We can then unpack the impact of these interventions on evidence use through a COM configuration as intermediate outcomes.

### 1.4 Why it is important to do this review

To facilitate learning from the dynamic body of research discussed above, Nduku et al.'s (2024) evidence map collected, organised, and visualised the available empirical, global evidence on interventions to support evidence‐informed policymaking across different policy sectors. Their map indicates that despite a global evidence base of more than 600 studies, there is a lack of evidence in a number of areas, including mechanisms working through raising awareness of EIDM. Empirical studies in this area typically focus on measuring intermediate outcomes of capability, motivation and opportunity to use evidence rather than attempting to measure actual change in evidence use. Additionally, they found synthesis gaps of the evaluation literature across the six mechanisms of change that they explored in the map. Previous systematic reviews in this area, such as Oliver et al. (2022), have not comprehensively identified, appraised, and synthesised the findings of this evidence base across different sectors. While the growth of EIDM and initiatives to encourage the use of evidence by policymakers is exciting, it is not clear which of these programmes and initiatives to support evidence use work best and why. This indicates there are several areas where research is needed, including to understand the contexts that can support evidence use in decision‐making and how to improve practice in this area.

The FCDO has approved funding for research on this topic, to address some of these research gaps and to inform their own practice and partners' EIDM practice. Topics of interest include when, where and how evidence is used in policymaking, the barriers and facilitators to evidence use, and what works to address these barriers and drive the uptake of evidence by decision‐makers. A systematic review of the existing evaluation literature on EIDM initiatives in policymaking is therefore needed as a first step to identify mechanisms, policies and programmes with promising evidence of effectiveness, and areas where further primary research would help consolidate the evidence base.

## 2 OBJECTIVES

We aim to collect, assess, and synthesise all the available empirical evidence on what works to support EIDM by policymakers. In doing so, we will aim to answer the following research questions:

(1) What are the impacts of interventions to support EIDM by policymakers?
(2) What are the factors which have influenced:
  (a) the impact of these interventions in low‐ and middle‐income countries (LMICs)?
  (b) their design and implementation in LMICs?

Answering these research questions will allow us to meet the following objectives:

- To estimate the overall impact and relative effectiveness of different evidence use interventions;
- To identify factors or configurations of factors that support or hinder the effectiveness of these interventions in LMICs;
- To identify gaps and areas to inform future primary research, particularly regarding the design, implementation, and evaluation of these interventions.

## 3 METHODS

We will conduct a systematic review of the existing empirical evidence (Gough et al., 2017) following guidelines for systematic reviews in social systems published by the Campbell Collaboration (2021). An ‘effectiveness plus’ (Snilstveit, 2012) systematic review with two parallel review modules will be conducted to answer the review questions on the extent to which interventions have been effective at supporting EIDM as well as what factors influence their impact. An effectiveness plus approach combines answering questions of what works with an equal emphasis on why and how it works, for whom, in what context, and so forth.

### 3.1 Criteria for considering studies for this review

Detailed inclusion criteria will determine what studies to include in this systematic review. We adopt the PICOS (Population, Intervention, Comparator, Outcome and Study design) framework to develop our inclusion criteria. The inclusion criteria define the precise characteristics of the studies that will be included in the review. All studies not meeting these criteria will be excluded from this review. As indicated above, we defined two sets of inclusion for research questions (1) and (2), respectively.

#### 3.1.1 Types of studies

For review question (1), we will include studies that assess the effects of interventions using experimental designs or quasi‐experimental designs (QEDs) with non‐random assignment that allow for causal inference, in line with Lwamba et al. (2021). Specifically, we include the following:

- RCTs, with assignment at individual, household, community, or other cluster level, and quasi‐RCTs using prospective methods of assignment such as alternation.
- Non‐randomised studies with selection on unobservables:
  (i). Regression discontinuity designs, where assignment is done on a threshold measured at pre‐test, and the study uses prospective or retrospective approaches of analysis to control for unobservable confounding.
  (ii). Studies using design or methods to control for unobservable confounding, such as natural experiments with clearly defined intervention and comparison groups, which exploit natural randomness in implementation assignment by decision makers (e.g., public lottery) or random errors in implementation, and instrumental variables estimation.

- Non‐randomised studies with pre‐intervention and post‐intervention outcomes data in intervention and comparisons groups, where data are individual‐level panel or pseudo‐panels (repeated cross‐sections), which use the following methods to control for confounding:
  (i). Studies controlling for time‐invariant unobservable confounding, including difference‐in‐differences, or fixed‐ or random‐effects models with an interaction term between time and intervention for pre‐intervention and post‐intervention observations; and
  (ii). Studies assessing changes in trends in outcomes over a series of time points (interrupted time series [ITS]), with or without contemporaneous comparison (controlled ITS), with sufficient observations to establish a trend and control for effects on outcomes due to factors other than the intervention (e.g., seasonality).

- Non‐randomised studies with control for observable confounding, including non‐parametric approaches (e.g., statistical matching, covariate matching, coarsened‐exact matching, propensity score matching) and parametric approaches (e.g., propensity‐weighted multiple regression analysis).

We will exclude all studies from the first review question that do not fall under any of the criteria defined above. Examples of excluded study types are: studies without a comparison group that use pre‐intervention and post‐intervention outcome data only, simulation studies that aim to predict the effect of a certain intervention, observational studies with no control for selection bias, life‐cycle analysis, process evaluations, and acceptability studies.

For review question (2), we will include a broad range of different study designs covering all empirical evaluation designs where ‘empirical’ is defined as a study applying a structured approach to both data collection and data analysis. This covers both more quantitative and more qualitative evaluation designs. This approach ensures that our systematic review includes a broad set of evidence on EIDM in LMICs in relation to our second review question. To be eligible for inclusion in our review question (2), studies must have explored the factors that have influenced the design, implementation, and impact of an applied EIDM intervention.

We will exclude the following types of evidence and study designs for either review question:

- Conceptual and theoretical studies, for example, frameworks and models, not based on empirical data.
- Opinion pieces, commentaries, and op‐eds.
- Studies merely reporting data, for example, monitoring data and administrative data without a structured analysis.
- Studies merely reporting an analysis of data without a structured approach to collect and quality assure the available data, for example, secondary analysis, big data.
- Studies reporting on both data collection and analysis, but where no EIDM intervention is assessed.
- We will also exclude certain types of publications:
  o Newspapers articles, blogs, opinion pieces, other social media.
  o Books and book chapters.
  o Honours thesis.

#### 3.1.2 Types of participants

We will include policy decision‐making behaviours and processes at the following levels:

1. individual
2. team
3. organisational (e.g., a government ministry/agency)
4. institutional (e.g., government‐wide, system‐specific)

Policy makers for the purpose of our review can refer to any individual working in a government department at any level of government (i.e. national, and sub‐national), including elected officials or civil servants that either could or should contribute to a policy process. It also covers individuals working in multilateral organisations, such as agencies and funds in the United Nations system, the World Bank and Inter‐American Development Bank. We do not place restrictions around the concept of ‘policy decision‐making behaviours and processes’ and this remains open to any form of decision‐making behaviours and processes, for example, starting or amending a policy, stopping a programme, changing the process for making decisions.

#### 3.1.3 Types of interventions

We will only include studies which evaluate or assess the effects of interventions aiming to increase policy makers' use of evidence. Such interventions can take many forms, for example, capacity‐building programmes to enhance decision‐makers' skills to access evidence or interventions aiming to connect decision‐makers and researchers (e.g., science cafés). All types of such EIDM interventions will be included and we will apply the mechanism structure introduced above to group interventions into categories for synthesis. We will include either single or multi‐component EIDM interventions, regardless of scale or intensity. This may include nudge type interventions if the nudge targets EIDM. If an intervention of any scale targets decision‐making more broadly it will not be included in the review, for example, Banuri et al. (2019).

Interventions must focus on policy makers' use of evidence. Evidence in this context is defined broadly as research‐based evidence (where we define research as a systematic investigative process employed to increase or revise current knowledge). In this systematic review, we will employ a broad conceptualisation of research that includes not only scientifically based research but extends to administrative data and statistics collected in the course of service and benefit provision. We will exclude studies that focus on the use of information more generally, for example, those that provide opinion surveys, citizen preference surveys and market research.

Interventions that focus on the uptake or implementation of evidence‐based practices or programmes (e.g., interventions to increase doctors' washing of hands) will be excluded. In the implementation science literature, there are many evaluations of interventions in which evidence use is understood as the adoption of an evidence‐based practice. The targeted behaviour change in this case is practitioners' implementation of a new practice, which happens to be evidence‐based. Gray et al. (2013) term this type of intervention as fostering the uptake of ‘empirically supported interventions (EIS)’, as opposed to interventions aiming to increase EIDM.

As described above, we will also exclude supply‐side interventions, such as financial incentives to produce more or better‐quality research. While supply‐side interventions are an important tool to enhance EIDM, for example, by increasing the policy‐relevance of research, the focus of this systematic review is on the direct use of evidence by policy decision‐makers (the art and science of using evidence). Supply‐side interventions in this context are outside the scope of this review as they do not directly target decision‐makers and rather aim to improve research undertaking itself (such as through funding channels) or to change researchers' behaviour. It is beyond the project's ability to assess the lengths of the causal chain from this change in research supply to decision‐makers' use of evidence. Interventions such as co‐production and engagement are, however, of relevance to this review in case they targeted decision‐makers' demand for evidence and were not narrowly focused on enhancing the supply of research.

Finally, studies that used experimentation or other methods solely to attempt to reveal policymakers' beliefs and biases when interpreting data or research studies or to understand approaches to decision‐making are out of scope of the review. However, if they also evaluated the effects of an intervention aiming to increase those policy makers' use of evidence or opportunity, motivation, or capability to do so, they will be included.

#### 3.1.4 Types of outcome measures

We will include studies that report data on either primary or intermediary evidence use outcomes. As indicated above, studies merely assessing the uptake of evidence‐based interventions or practices (e.g., washing of hands) will be excluded from our review. Essentially, this approach is synonymous with evaluating a common adoption of a new practice and its performance. If studies aim to evaluate an intervention to increase evidence use, outcomes must be structured to capture changes in evidence use, that is the practice of EIDM (Thompson et al., 2007). In our systematic review, the targeted behaviour change is the use of evidence rather than the adoption of individual evidence‐based practices. Unfortunately, this distinction is often not made explicit in the wider literature. For the purpose of this systematic review, we will resort to analysing the reported outcome measures in the included studies to ensure that the outcomes meet our definition of EIDM.

##### Primary outcomes

Our systematic review focuses on two primary outcomes of interest.

###### Evidence use

This refers to the practice of EIDM. We define EIDM as:

‘a process whereby multiple sources of information, including the best available research evidence, are consulted before making a decision to plan, implement, and (where relevant) alter policies, programmes, and other services’ (Langer et al., 2016).

EIDM or evidence use as an outcome is therefore not the alignment of the policy content with the available evidence‐base, but the extent to which the policy formulation process was informed by evidence. Or, to put it differently, EIDM as an outcome implies that the decision‐maker has engaged with the evidence and acted upon it in some way. Acting upon evidence does not necessarily mean that it has been used to inform policy or practice developments. It could simply mean that the findings were considered during policy discussions. This suggests that a policy decision where evidence was considered, even if not fully integrated, should still be regarded as evidence‐informed.

Furthermore, there are different ways in which evidence can inform a decision. Based on Weiss' (1979) typology of evidence use, two types of evidence use apply to this study:

*Instrumental evidence use* is a direct use of evidence, knowledge, and insights. It refers to the concrete application of evidence, such as in the taking of specific policy decisions or implementation of practice interventions.

*Conceptual evidence use* highlights evidence's enlightenment function. This is when evidence influences how policymakers and practitioners think about issues, problems, or potential solutions. Evidence findings may change their opinion but not necessarily a particular action.

Relevant indicators for the primary outcomes of evidence use include but are not limited to: research evidence being referenced in policy documents, or utilised in programme or guideline development; EIDM indicators, for example, Global EIDM index (Dobbins et al., 2009); evidence of decision‐makers' behaviour change, for example, accessing, appraising, considering evidence as part of a decision‐maker's daily practice.

Lastly, evidence use for policy decision‐making can occur at two stages: first, at the policy design stage and, second, at the policy implementation stage. For evidence use to influence socio‐economic development, both policy design *and* implementation have to be effective and equitable. Where possible, we will code evidence use outcomes for the respective stage in the policy cycle. For the meta‐analysis, we will focus on evidence use as the primary layer of analysis, with policy design and policy implementation being variables for potential sub‐group analysis.

###### Socio‐economic impact^2^

This refers to the impact of an increased use of evidence on development indicators. For example, a sustained practice of EIDM can be associated with better health outcomes such as reduced mortality rates. Likewise, evidence use can affect educational outcomes such as increased test scores and grade pass rates. Indicators of development impact are not prespecified and can be cross‐sectoral covering all 17 Sustainable Develoment Goals.

##### Secondary outcomes

*Intermediary outcomes*: This refers to outcomes assessing intermediate conditions and activities that enhance the likelihood of decision‐makers using evidence. As above, intermediate outcomes were separated using Michie et al.'s (2011) COM model of behaviour change (Table 2). These may be measured through self‐report by policymakers but could also be measured through researchers' perceptions.

**Table 2:** Intermediary outcomes.

1. Capability to use evidence	This refers to decision‐makers having the required psychological and physical capacity to engage in EIDM. It includes having the necessary knowledge and skills. Indicators of this intermediate outcome include test scores evaluating respondents' knowledge of EIDM concepts as well as critical appraisal skills.
2. Motivation to use evidence	This refers to the brain processes that energise and direct behaviour, not just goals and conscious decision‐making. It includes habitual processes, emotional responding, as well as analytical decision‐making. Indicators of this intermediate outcome include attitudes towards evidence or decision‐makers' reported intention to use evidence.
3. Opportunity to use evidence	This refers to all the factors that lie outside the decision‐makers' control that make the EIDM possible or prompt it. Indicators of this intermediate outcome include access to evidence databases or organisational processes for EIDM.

#### 3.1.5 Types of settings

For research question (1), we will include studies in any country.

For research question (2), we will only include studies that assess an EIDM intervention conducted in LMICs as defined by the World Bank classification of economies.^3^ Where studies are conducted in multiple countries or regions including HIC countries or regions, their empirical data needs to be disaggregated for LMICs or regions in order for the study to be included.

#### 3.1.6 Other criteria

Both academic and Grey Literature are eligible for inclusion in the review. We will only consider English publications for the review. There is no limitation on the publication date of the study.

### 3.2 Search methods for identification of studies

#### 3.2.1 Electronic searches

For this review, we will update the search of an existing evidence and gap map that fully overlaps with the review's scope and was conducted in January 2023 (Nduku et al., 2024). The search strategy followed by Nduku et al. (2024) designed a scientific and exhaustive search for evidence with the help of an information scientist. The search strategy is based on three pillars: (i) an exhaustive and replicable search of the indexed academic literature; (ii) an in‐depth search of available Grey literature sources; and (iii) a forward and backward search including key informants.

First, we will update the searches for all academic evidence on EIDM in the eight academic databases including PubMed, Web of Science, Scopus, and EBSCO Host (ERIC, PsycINFO, Business Source Complete, Communication and Mass Media complete, and Political Science Complete). A combination of key terms was adopted and included *evidence use terms* (e.g., ‘evidence use’ OR ‘evidence utilisation’ ‘research use’ OR ‘research utilisation’ OR ‘knowledge use’ OR ‘knowledge utilisation’ OR ‘evaluation use’ OR ‘evaluation utilisation’); *evidence into action terms* (e.g., ‘evidence broker*’ OR ‘evidence champion*’ OR ‘research broker*’ OR ‘research champion*’); *evidence‐informed decision‐making terms* (e.g., ‘evidence‐based’ OR ‘evidence‐informed’); and *policy‐ and decision‐making terms* (e.g., policy OR policies OR decision* OR ‘decision‐making’ OR ‘decision making’ OR ‘policy‐making’ OR ‘policy making’ OR policymaking).

#### 3.2.2 Searching other resources

Second, we will also carry out search updates in grey literature sources such as websites of specialist organisations to find studies meeting our inclusion criteria that are outside of the indexed academic literature and that were published since the previous search was conducted, from January 2023 to February 2024. These searches utilise key words only given the websites' limited search capabilities. Third, we will conduct backward and forward citation‐tracking of key authors and publications on Google Scholar for all newly identified studies. Appendix S1A indicates the comprehensive search strings to be applied whilst Supporting Information: Appendix S1B presents a full list of all the academic and Grey literature search sources.

### 3.3 Data collection and analysis

#### 3.3.1 Description of methods used in primary research

In relation to the first review question, the systematic review will only include primary studies that measure the effects of interventions and whose design can reliably attribute observed effects to the applied interventions, specifically RCTs and QEDs. Individual effects will be synthesised into overall estimates of treatment effects using statistical meta‐analysis where possible. If we are unable to undertake statistical meta‐analysis due to insufficient studies or heterogeneity in the included study intervention and outcomes, we will provide a narrative discussion of the effect sizes in the included studies. For the second review question, we will include any form of empirical evaluation of an evidence use intervention that addresses research question 2 and apply qualitative evidence synthesis approaches to synthesise the results of these evaluation studies.

#### 3.3.2 Criteria for the determination of independent findings

Complex data structures are a common occurrence in meta‐analyses of impact evaluations. There are numerous scenarios through which these complex structures with dependent effect sizes might occur. For example, there could be several publications that stem from one study, or several studies based on the same data set. Some studies might have multiple treatment arms that are all compared to a single control group. Other studies may report outcome measurements from several time points or use multiple outcome measures to assess related outcome constructs. All such cases yield a set of statistically dependent effect size estimates (Borenstein et al., 2009).

The research team will assess the extent to which relationships exist across the studies included in the review and avoid double counting of identical evidence by linking papers before data analysis. Where we have several publications reporting on the same effect, we will use effect sizes from the most recent publication. We will utilise information provided in studies to support these assessments, such as sample sizes, programme characteristics and key implementing and/or funding partners.

We will extract effects reported across different outcomes or subgroups within a study, and where information is collected on the same programme for different outcomes at the same or different periods, information on the full range of outcomes over time will be extracted. Where studies report effects from multiple model specifications, we will adopt the author's preferred model specification. If this is not stated or is unclear, we will extract effect data from the most precise model (e.g., the model with the smallest standard error). Where studies report multiple outcomes or evidence according to sub‐groups of participants, we will record and report data on relevant sub‐groups separately. Further information on criteria for determining independent effect sizes is presented below.

We will deal with dependent effect sizes through data processing and selection techniques, that utilise several criteria to select one effect estimate per study. For studies with outcome measures at different time points, we will follow De La Rue et al. (2017) and synthesise outcomes measured immediately after the intervention (defined as 1‐6 months) and at follow‐up (longer than 6 months) separately. If multiple time points exist within these periods, we adopt the most recent measure. We anticipate that some of the interventions that we will include in our review would be ongoing programmes and the follow‐up would, therefore, reflect duration in a programme rather than time since the intervention. When such studies report outcome measures at different time points, we will identify the most common follow‐up period and include the follow‐up measures that match this most closely in the meta‐analysis. When studies include multiple outcome measures to assess related outcome constructs, we will follow Macdonald et al. (2012) and select the outcome that appears to reflect the construct of interest most accurately without reference to the results.

If studies include multiple treatment arms with only one control group and the treatments represent separate treatment constructs, we calculate the effect size for treatment A versus control and treatment B versus control and include them in separate meta‐analyses according to the treatment construct. Where different studies report on the same programme but use different samples (e.g., from different regions, or separately for men and women) we will include both estimates, treating them as independent samples, provided effect sizes are measured relative to separate control or comparison groups.

#### 3.3.3 Selection of studies

Review management software (EPPI Reviewer 4) will be used to manage the entire review process. All potentially relevant citations gathered from the academic sources above will be imported into EPPI Reviewer 4. They will undergo a detailed screening process to be assessed for eligibility using the inclusion criteria highlighted above, and decisions made about each citation will be recorded on the same platform. Search results from organisational websites and the citation searches will be captured in MS Word and only studies deemed to be relevant for the review will be transferred to EPPI Reviewer 4. Studies that are not already on EPPI Reviewer will be captured manually on the software. Before proceeding with screening, all duplicates of titles will be excluded from the review using the duplicate control function on EPPI reviewer 4.

We will test reviewer bias (interrater reliability) at the start of each stage of the screening process using a Kappa analysis (Collaboration for Environmental Evidence CEE, 2013). Two reviewers will screen a common random sample of 10% of abstracts. The level of agreement between the number of articles rejected or accepted by the Kappa statistic will be calculated on a scale that ranges from 1 (perfect agreement) and ‐1 (strong disagreement). The individual screening will only be permissible once a Kappa statistic score of 0.85 or above is achieved. A third‐party arbitrator will resolve any disagreements at both stages of the screening process. The screening process will be reported using a PRISMA flow chart.

#### 3.3.4 Data extraction and management

We will use a predefined data extraction tool to extract data systematically and transparently from the included primary studies. This data extraction tool is presented in Appendix S1C and will be migrated into EPPI‐Reviewer 4 to extract information that is required for the evidence synthesis. The data will be entered directly into the EPPI‐Reviewer database and full‐text reports will be examined and studies coded on variables related to:

(a) Descriptive data including authors, publication date and type, as well as other information to characterise the study including country, type of intervention, outcome, population, and context.
(b) Information on intervention design, implementation fidelity, factors that influenced design, implementation and impact, and possible programme mechanisms.

To ensure consistency of coding quality, two reviewers will pilot the data extraction tool, working independently on a random sample (10%) of eligible studies selected to test the tool on the complete range of the included impact evaluation designs and methods. The process will be repeated until and very high level of consistency, defined by a minimum Kappa statistic score of 0.85, in the reviewer's application of codes is attained and the tool will be deemed final. Following the piloting stage, the remaining studies will be coded by individual reviewers, with a subset of these full texts being coded by different combinations of two reviewers independently extracting information from each study and then comparing their decisions. Any uncertainties or disagreements will be resolved via discussion, with a third‐party arbitrator resolving any outstanding disagreements.

A summary of findings table of all included studies will be provided to highlight key data extracted from across the included studies.

Data extraction for the purpose of effect size calculation is discussed below.

#### 3.3.5 Assessment of risk of bias

We will conduct two separate critical appraisal processes in our systematic review: the first will entail a risk of bias assessment of the included impact evaluations in review question (1) while the second will entail a quality assessment of the different types of evaluations included in review question (2).

For review question (1), we will apply a critical appraisal tool to assess the impact of bias on the trustworthiness of primary impact evaluations included in the systematic review. Trustworthiness refers to the confidence of the review team that the findings reported in the included studies used for the synthesis were rigorous and credible. To assess the risk of bias of the primary studies, we will adapt the Cochrane risk of bias tool for randomised and non‐randomised studies (Sterne et al., 2016), which we have previously used and adapted in international development reviews (Stewart et al., 2015; Langer et al., 2018). The tool is provided in Appendix S1D. Sterne and colleagues used a domain‐based risk of bias tool covering the following six indications of trustworthiness: (i) selection bias; (ii) confounding bias; (iii) bias due to departures from applied interventions; (iv) bias due to missing data; (v) bias due to measurement of outcomes; and (vi) bias due to selection of the reported result. Each domain of bias will receive a low, moderate, high or critical risk of bias rating, allowing for a transparent calculation of the overall risk of bias score for each study. Studies with a critical risk of bias will be included in the review but excluded from the synthesis. The risk of bias tool will be piloted using a similar approach to that used for the piloting of the data extraction tool. Two reviewers will independently assess each study and then come together to compare their decisions. Where these reviewers disagree about the risk of bias rating for a particular study, a third reviewer will be consulted.

#### 3.3.6 Qualitative critical appraisal

For review question (2), we will assess the quality of included qualitative evaluations and process evaluations using a mixed‐methods appraisal tool developed by Langer and colleagues (2018) and applied in Snilstveit et al. (2019). This tool is provided in Appendix S1E. This tool builds on the Critical Appraisal Skills Programme checklist (Critical Appraisal Skills Programme CASP, 2006) and Pluye et al.'s (2011) mixed‐methods appraisal tool and is provided in Appendix S1E. Our appraisal tool will make judgements on the adequacy of reporting, data collection, presentation, analysis and conclusions drawn. The appraisal assesses the quality of the included studies for review question (2) using six appraisal domains:

1. The defensibility of the applied research design to answer the research question under investigation.
2. The defensibility of the selected research sample and the process of selecting research participants.
3. The rigour of the technical research conduct, including the transparency of reporting.
4. The rigour of the applied analysis and credibility of study's claims given the nature of the presented data.
5. The consideration of the study's context (for qualitative studies only).
6. The reflexivity of the reported research (for qualitative studies only).

We will filter out studies of particularly low quality at this stage, using a fatal flaw approach following Dixon‐Woods et al. (2005). Studies that do not meet either criterion of appraisal domains 1–4 above will be excluded from the synthesis. That is, they will be included in the review, and we will report on the studies' descriptive data, for example, applied intervention. However, no research findings will be extracted from these studies to feed into the review's synthesis. Each appraisal domain will be assessed from a scale of critical trustworthiness to low, medium and high trustworthiness. An overall critical appraisal judgement per study will be allocated using a numerical threshold of the appraised quality domains (Appendix S1E).

#### 3.3.7 Measures of treatment effect

Quantitative data for outcome measures, including outcome descriptive information, sample size in each intervention group, outcomes means and standard deviations (SDs), and test statistics (e.g., *t*‐test, *F*‐test, *p*‐values, 95% confidence intervals) will be extracted using Excel (see the preliminary data extraction form in Appendix S1F). Effect size data will be stored, and any necessary cleaning will be conducted in Excel. Following the screening and descriptive data extraction process of ensuring consistency in coding quality, two reviewers will pilot the effect size data extraction tool, working independently on a random sample (10%) of included studies to test the tool across a range of the included impact evaluation designs and methods. We aim to achieve a minimum Kappa statistic score of 0.85 following a round of repeating the process for the tool to be finalised. After the piloting stage, the remaining studies will be coded by individual reviewers and all data extracted will be checked by a third reviewer.

An effect size expresses the magnitude (or strength) and direction of the relationship of interest (Borenstein et al., 2009; Valentine et al., 2015). We will extract data from each study to calculate standardised effect sizes for cross‐study comparison wherever possible. For continuous outcomes comparing group means in a treatment and control group, we will calculate the standardised mean difference (SMDs), or Cohen's *d*, its variance and standard error using formulae provided in Borenstein et al. (2009). An SMD is a difference in means between the treatment and control groups divided by the pooled SD of the outcome measure. Cohen's *d* can be biased in cases where sample sizes are small. Therefore, in all cases we will adjust using Hedges' method, adjusting Cohen's *d* to Hedges' *g* using the following formula (Ellis, 2010):

$$g≅d(1-\frac{3}{4(n_{1}+n_{2})-9}).$$

We will choose an appropriate formula for effect size calculations in reference to, and dependent upon, the data provided in included studies. For example, for studies reporting means (*X*) and pooled SD for treatment (*T*) and control or comparison (*C*) at follow up only:

$$d=\frac{x_{Tp+1}-x_{Cp+1}}{\mathrm{SD}}.$$

If the study does not report the pooled SD, it is possible to calculate it using the following formula:

$${\mathrm{SD}}_{p+1}=\sqrt{\frac{(n_{Tp+1}-1){\mathrm{SD}}_{\mathrm{Tp}+1}^{2}+(n_{Cp+1}-1){\mathrm{SD}}_{\mathrm{Cp}+1}^{2}}{n_{\mathrm{Tp}+1}+n_{\mathrm{Cp}+1}-2}},$$

where the intervention is expected to change the SD of the outcome variable, we will use the SD of the control group only.

For studies reporting means ($\underline{X})$ and SDs for treatment and control or comparison groups at baseline (*p*) and follow up (*p* + 1):

$$d=\frac{{∆\underline{X}}_{p+1}-{∆\underline{X}}_{p}}{{\mathrm{SD}}_{p+1}}.$$

For studies reporting mean differences ($∆\underline{X})$ between treatment and control and SD at follow up (*p* + 1):

$$d=\frac{{∆\underline{X}}_{p+1}}{{\mathrm{SD}}_{p+1}}=\frac{{\underline{X}}_{\mathrm{Tp}+1}-{\underline{X}}_{\mathrm{Cp}+1}}{{\mathrm{SD}}_{p+1}}.$$

For studies reporting mean differences between treatment and control, standard error (SE) and sample size (*n*):

$$d=\frac{{∆\underline{X}}_{p+1}}{\mathrm{SE}\sqrt{n}}.$$

As primary studies have become increasingly complex, it has become commonplace for authors to extract partial effect sizes (e.g., a regression coefficient adjusted for covariates) in the context of meta‐analysis. For studies reporting regression results, we will follow the approach suggested by Keef and Roberts (2004) using the regression coefficient and the pooled SD of the outcome. Where the pooled SD of the outcome is unavailable, we will utilise regression coefficients and standard errors or *t*‐statistics to do the following, where sample size information is available in each group:

$$d=t\sqrt{\frac{1}{n_{T}}+\frac{1}{n_{C}}},$$

where *n* denotes the sample size of the treatment group and control. We will use the following where only the total sample size information (*N*) is available, as suggested in Polanin et al. (2016):

$$d=\frac{2t}{\sqrt{N}}{\mathrm{Var}}_{d}=\frac{4}{N}+\frac{d^{2}}{4N}.$$

We will calculate the *t*‐statistic (*t*) by dividing the coefficient by the standard error. If the authors only report confidence intervals and no standard error, we will calculate the standard error from the confidence intervals. If the study does not report the standard error but reports *t*, we will extract and use this as reported by the authors. In cases in which significance levels are reported rather than *t* or SE (b), then *t* will be imputed as follows:

Prob>0.1:t=0.5

0.1≥Prob>0.05:t=1.8

0.05≥Prob>0.01:t=2.4

0.01≥Prob:t=2.8

Where outcomes are reported in proportions of individuals, we will calculate the Cox‐transformed log odds ratio effect size (Sánchez‐Meca et al., 2003):

$$d=\frac{\ln(\mathrm{OR})}{1.65},$$

where OR is the odds ratio calculated from the two‐by‐two frequency table.

Where outcomes were reported based on proportions of events or days, we will use the standardised proportion difference effect size:

$$d=\frac{p_{T}-p_{C}}{\mathrm{SD}(p)},$$

where *p*_*t*_ is the proportion in the treatment group and *p*_*c*_ the proportion in the comparison group, and the denominator is given by:

$$\mathrm{SD}(p)=\sqrt{p\;(1-p)},$$

where *p* is the weighted average of *p*_*c*_ and *p*_*t*_:

$$p=\frac{n_{T}\;p_{T}+n_{C}\;p_{C}}{n_{T}+n_{C}}.$$

An independent reviewer will evaluate a random selection of 10% of effect sizes to ensure that the correct formulae will be employed in effect size calculations. In all cases after synthesis, we will convert the pooled effect sizes to commonly used metrics such as percentage changes and mean differences in outcome metrics typically used (e.g., weight in kg) whenever feasible.

##### Unit of analysis issues

Unit of analysis errors can arise when the unit of allocation of a treatment is different from the unit of analysis of effect size estimate, and this is not accounted for in the analysis (e.g., by clustering standard errors at the level of allocation). We will assess studies for unit of analysis errors (The Campbell Collaboration, 2021), and where they exist, we correct for by adjusting the standard errors according to the following formula (Higgins & Thomas, 2020; Hedges, 2011; Waddington et al., 2012):

$$SE(d)\prime =SE(d)*\sqrt{1\;+(m-1)c},$$

where *m* is the average number of observations per cluster and *c* is the intra‐cluster correlation coefficient. Where included studies used robust Huber‐White standard errors to correct for clustering, we will calculate the standard error of *d* by dividing *d* by the t‐statistic on the coefficient of interest.

#### 3.3.8 Dealing with missing data

We plan to write to authors of studies included to address research question 1 to obtain any data missing from these studies.

#### 3.3.9 Meta‐analysis

To address research question 1, we will aim to conduct statistical meta‐analyses of studies that are assessed to be sufficiently similar and only combine studies using meta‐analysis when we identify two or more effect sizes using a similar outcome construct and where the comparison group stated is judged to be similar across the two (c.f. the approach taken by Wilson et al. [2011]). We will combine studies in the same analysis when they evaluate the same mechanism type, or use the same combination of mechanisms (e.g., access and interaction), and the same outcome type (i.e., evidence use, the three intermediate outcome categories or socioeconomic impact). We plan to use the metafor package in R to undertake meta‐analysis (Viechtbauer, 2010). Where there are too few studies or included studies are considered too heterogeneous in terms of interventions or outcomes, we will discuss the individual effect sizes along the causal chain. We anticipate heterogeneity across studies, and so we will adopt inverse‐variance weighted, random effects meta‐analytic models (Higgins & Thompson, 2002) to account for this.

We will conduct separate analyses for the major outcome categories for each mechanism where possible: that is, by evidence use, socio‐economic impact and the three categories of intermediate outcomes (capability, motivation, opportunity to use evidence).

#### 3.3.10 Subgroup analysis and investigation of heterogeneity

We anticipate that we will have a limited number of included impact evaluations, and therefore will be unlikely to be able to undertake moderator analysis to try to explain variations in effect sizes. However, we are interested in whether the following variables could explain variation in effect sizes:

– Interventions targeted at local versus national levels of government.
– Sector
– Geography
– Socioeconomic status
– Single versus multicomponent interventions
– Stage of the policy cycle (e.g., policy design, policy implementation)

If we are able to undertake moderator analyses, it will be reported in a tabular format below each meta‐analysis, calculated using meta‐regression.

To visibly examine variability in the effect size estimates, we will use forest plots to display the estimated effect sizes from each study along with their 95% confidence intervals. Subsequently, and acknowledging the limitations of quantification of heterogeneity and the different strengths of statistical approaches, the following test for heterogeneity will be conducted: calculation of the *Q*‐statistic as a statistical test of heterogeneity (Hedges & Olkin, 2014); and calculation of the *I* ^2^ and *τ* ^2^ statistic to provide estimates of the magnitude of the variability across study findings caused by heterogeneity (Borenstein et al., 2009; Higgins & Thompson, 2002; Higgins, 2003).

#### 3.3.11 Sensitivity analysis

To test the robustness of the results of the meta‐analysis, a number of sensitivity analyses will be conducted. Broadly, this involves collecting data on and assessing the sensitivity of findings to (i) the methods of the primary studies and (ii) the methods of the review. We anticipate that the included studies will vary methodologically and will therefore conduct sensitivity analyses to examine the influence of these variations on the summary measures, to offer possible explanations for the differences between studies when interpreting the results. We will examine whether the results were sensitive to study design, the risk of bias associated with the study, the degree of missing/incomplete data, and the way outcomes are measured and the timing at which they are measured. The main objective of the sensitivity analysis is to serve as a visual tool that allows informal comparisons to determine whether the results of our meta‐analyses are sensitive to the methodological decisions of the review team.

#### 3.3.12 Treatment of qualitative research

To address research question 2, we intend to apply thematic synthesis as our preferred approach to the qualitative evidence synthesis. Thematic synthesis depends on the availability of sufficient in‐depth qualitative studies and empirical primary data reported across the identified evidence‐base and linked to groups of interventions and outcomes along the review's logic model. This objective of this synthesis approach is to identify analytical themes on factors that have influenced the design, implementation and impact of the interventions of interest. This will complement any statistical moderator analysis or meta regression we are able to run, although we do not expect to have sufficient studies to run such analysis. Following Thomas and Harden's (2008) thematic synthesis, we will apply inductive coding techniques to first identify common descriptive themes based on the reported findings of the primary studies. We will use EPPI‐Reviewer's in‐built qualitative synthesis coding software to illustrate the link between the inductive codes in the primary studies and the identified descriptive themes. In a second step, following the identification of descriptive themes, these then will be configured into higher level analytical themes, which present the results of the thematic synthesis. Again, this configuration from descriptive to analytical themes is conducted in EPPI‐Reviewer and we will produce an overview table of both types of themes and their linkages for transparency in this final synthesis step. The process of configuring descriptive and analytical themes from the inductive coding will apply the same consistency checks as the general data extraction process outlined above. That is, two reviewers will pilot the data extraction tool, working independently on a random sample (10%) of eligible studies selected to test the tool on the complete range of types of studies. The process will be repeated until there is a very high level of consistency, defined by a minimum Kappa statistic score of 0.85.

The process of generating inductive codes, descriptive themes, and final analytical themes will be configured around the analytical lenses derived from the research question 2 of this review, detailed below. We will synthesise the extracted qualitative evidence regarding the interplay of four groups of factors with the intervention effect, outcome, or impact.

I. Intervention design: any factor that is related to the design and planning of the applied intervention. Design and planning of an intervention refers to the blueprint or schedule of the intervention and will typically outline what components the intervention consists of and in what sequence they will be applied.

II. Intervention implementation: any factor that is related to the implementation of the intervention in practice. This refers to variables that emerge while the intervention is applied and are usually not known in advance. For example, there may be contextual factors that have influenced a lack of attendance or uptake.

IV. Context: any factors related to external influences beyond the programme's control that affect intervention design, implementation or impact. This can refer to political factors such as types of governance, societal factors such as norms, economic factors such as a recession, and cultural factors such as beliefs.

V. Population characteristics: any factors related to the population targeted by the intervention or the population in which the effects are measured (in cases where these differ).

## CONTRIBUTION OF AUTHORS

The review is being led by a team at the Pan‐Africa Collective for Evidence (PACE): Laurenz Mahlanza‐Langer, Promise Nduku, John Ategeka, Tanya Mdlalose, Ruvimbo Nhandara.

The review is being supported by a team at 3ie: Jennifer Stevenson, Shannon Shisler, Suvarna Pande.

John Young at INASP is providing subject expertise.

## DECLARATIONS OF INTEREST

There are no reported conflicts of interest on this review.

## PRELIMINARY TIMEFRAME

The planned time frame for this systematic review is as follows:

- Protocol development: January–February 2024
- Search update, data extraction, critical appraisal, synthesis: February–June 2024
- Draft and Final Report: May–August 2024

## PLANS FOR UPDATING THE REVIEW

The authors do not have plans to update the review at this time.

## SOURCES OF SUPPORT

### External sources

Funding for this systematic review was provided by the Foreign and Commonwealth Development Office (FCDO) through the Research Commissioning Centre (RCC). The RCC is a virtual hub established to effectively manage select development and diplomacy research by the Research and Evidence Directorate (RED) of the FCDO. Led by the International Initiative for Impact Evaluation (3ie), the University of Birmingham and a consortium of UK and global research partners, the RCC aims to commission different types of high‐quality research in key priority areas.

## Supporting information

Supporting information.

## Footnotes

1
*Capability* is defined as the individual's psychological and physical capacity to engage in the activity concerned. It includes having the necessary knowledge and skills. *Motivation* is defined as all those brain processes that energise and direct behaviour, not just goals and conscious decision‐making. It includes habitual processes, emotional responding, as well as analytical decision‐making. *Opportunity* is defined as all the factors that lie outside the individual that make the behaviour possible or prompt it (Michie et al., 2011).

2 We do not expect many studies covering the pathway from evidence use to socio‐economic impact. Arguably, this last step in the causal chain is beyond the control of the applied EIDM intervention and as a result the applied intervention should not be assessed against outcome.

3
https://blogs.worldbank.org/opendata/new-world-bank-country-classifications-income-level-2022-2023.
