# Supplementary material for: Protocol: What works to increase the use of evidence for policy decision‐making: A systematic review
Source: Campbell Syst Rev. 2024 Nov 22;20(4):e1435. doi: 10.1002/cl2.1435 (PMC11582683; doi:10.1002/cl2.1435)
Supplement: Supplementary file 1 — Supporting information. [file CL2-20-e1435-s001.docx]

**Appendices**

**Appendix A: Search strategy**

**Overall Search Term:**

**(#1 OR #2 OR #3) AND #4**

**#1 Comprehensive evidence use terms**

“evidence use” OR “evidence utilisation” OR “evidence utilization” OR “evidence dissemination” OR “evidence diffusion” OR “evidence uptake” OR “evidence mobilisation” OR “evidence mobilization” OR “evidence application” OR “evidence translation” OR “evidence transfer” OR “evidence adoption” OR “evidence sharing” OR “evidence implementation” OR “evidence exchange”

OR

“research use” OR “research utilisation” OR “research utilization” OR “research dissemination” OR “research diffusion” OR “research uptake” OR “research mobilisation” OR “research mobilization” OR “research application” OR “research translation” OR “research transfer” OR “research adoption” OR “research sharing” OR “research implementation” OR “research exchange”

OR

“knowledge use” OR “knowledge utilisation” OR “knowledge utilization” OR “knowledge dissemination” OR “knowledge diffusion” OR “knowledge uptake” OR “knowledge mobilisation” OR “knowledge mobilization” OR “knowledge application” OR “knowledge translation” OR “knowledge transfer” OR “knowledge adoption” OR “knowledge sharing” OR “knowledge implementation” OR “knowledge exchange”

OR

“evaluation use” OR “evaluation utilisation” OR “evaluation utilization” OR “evaluation dissemination” OR “evaluation diffusion” OR “evaluation uptake” OR “evaluation mobilisation” OR “evaluation mobilization” OR “evaluation application” OR “evaluation translation” OR “evaluation transfer” OR “evaluation adoption” OR “evaluation sharing” OR “evaluation implementation” OR “evaluation exchange”

**#2 Evidence into Action terms (supplement)**

“evidence broker*” OR “evidence champion*” OR “evidence into action” OR “evidence into practice” OR “evidence into policy” OR “evidence to action” OR “evidence to practice” OR “evidence to policy”

“research broker*” OR “research champion*” OR “research into action” OR “research into practice” OR “research into policy” OR “research to action” OR “research to practice” OR “research to policy”

“knowledge broker*” OR “knowledge champion*” OR “knowledge into action” OR “knowledge into practice” OR “knowledge into policy” OR “knowledge to action” OR “knowledge to practice” OR “knowledge to policy”

“evaluation broker*” OR “evaluation champion*” OR “evaluation into action” OR “evaluation into practice” OR “evaluation into policy” OR “evaluation to action” OR “evaluation to practice” OR “evaluation to policy”

**#3 Evidence-informed decision-making terms**

(“evidence-based” OR “evidence-informed”) AND (policy OR policies OR decision* OR “decision-making” OR “decision making” OR “policy-making” OR “policy making” OR policymaking)

OR

“data use” OR “research impact” OR “evidence ecosystem” OR “evidence system” OR “knowledge system” OR “evidence movement” OR “evidence agenda”

OR

(“use of evidence” OR “use of research” OR “use of knowledge” OR “use of evaluation” OR “uptake of research” OR “uptake of evidence” OR “uptake of knowledge” OR “uptake of evaluation”)

**Appendix B: Search sources**

1. Electronic academic databases

| **Database** | **Search results** |
| --- | --- |
| Healthcare: |  |
| 1. Medline/PubMed |  |
| Broad Social Sciences: |  |
| 1. Web of Science (Science Citation Index Expanded, Social Science Citation Index, Emerging Sources Citation Index) |  |
| 1. Scopus |  |
| All searched via EbscoHost |  |
| Education: |  |
| 1. ERIC |  |
| Psychology/Behavioural Sciences: |  |
| 1. PsycINFO |  |
| Organisational: |  |
| 1. Business Source Ultimate |  |
| Communication: |  |
| 1. Communication and Mass Media complete |  |
| Political Science: |  |
| 1. Political Science Complete |  |

1. Grey literature sources

| **Website** | **URL** |
| --- | --- |
| 1. Africa Centre for Evidence | https://africacentreforevidence.org/ |
| 1. Africa Evidence Network | https://www.africaevidencenetwork.org/en/ |
| 1. BCURE | <https://bcureglobal.wordpress.com> |
| 1. African Institute for Development Policy (AFIDEP) | https://www.afidep.org/ |
| 1. African Academy of Sciences | https://www.aasciences.africa/ |
| 1. Africa Centre for Systematic Reviews and Knowledge Translation (ACSRKT) | https://chs.mak.ac.ug/afcen/ |
| 1. Zimbabwe Evidence Informed Policy Making Network (ZEIPNET) | https://www.zeipnet.co.zw/ |
| 1. PACKS-Africa | https://www.packs-africa.org/ |
| 1. Africa Cabinet Network | http://www.cabinetgovernment.net/ |
| 1. The Overseas Development Institute (ODI) | https://odi.org/en/ |
| 1. International Network for the Availability of Scientific Publications (INASP) | https://www.inasp.info/ |
| 1. 3ie | https://www.3ieimpact.org/evidence-hub |
| 1. WACIE | https://www.3ieimpact.org/our-work/west-africa-capacity-building-and-impact-evaluation |
| 1. International Network for Government Science Advice (INGSA) | https://www.ingsa.org/ |
| 1. South African Department of Planning, Monitoring and Evaluation (DPME) | https://www.dpme.gov.za/Pages/default.aspx |
| 1. Twende Mbele | https://twendembele.org/ |
| 1. Human Sciences Research Council (HSRC) | http://www.hsrc.ac.za/en |
| 1. Council for Scientific and Industrial Research (CSIR) | https://www.csir.co.za/ |
| 1. University of Cape Town (UCT) | https://www.uct.ac.za/ |
| 1. Makerere University | https://www.mak.ac.ug/ |
| 1. SDG Hub | https://sdg.iisd.org/ |
| 1. eBase Africa | https://www.ebaseafrica.org/ |
| 1. Centre for the Development of Best Practices in Health (Cameroon) | http://www.cdbph.org/index.php/en/ |
| 1. Ethiopian Public Health Institute (Ethiopia) | http://www.ephi.gov.et/ |
| 1. Ebonyi State University (Nigeria) | https://www.ebsu.edu.ng/# |
| 1. Ministry of Health (Burkina Faso) | https://www.sante.gov.bf/accueil |
| 1. School of Medicine, Faculty of Medicine, University of Antioquia (Colombia) | https://bit.ly/2hHkosq |
| 1. Veredas Institute (Brazil) | https://www.veredasinstitute.com/ |
| 1. Foundation for Scientific and Technological Development in Health (FIOTEC) | https://www.fiotec.fiocruz.br/en/access-to-information |
| 1. Ministry of Health (Chile) | https://www.minsal.cl/ |
| 1. Centre of Studies and Research, Ministry of Health (Oman) | https://mohcsr.gov.om/ |
| 1. Lanzhou University | https://en.lzu.edu.cn/ |
| 1. Knowledge to Policy Center, American University of Beirut (Lebanon) | https://www.aub.edu.lb/k2p/Pages/default.aspx |
| 1. EPPI Centre, UCL Institute of Education, University College London (United Kingdom) | https://eppi.ioe.ac.uk/cms/ |
| 1. Results for All | https://results4america.org |
| 1. Jimma University Ethiopia | https://www.ju.edu.et/ |
| 1. Partnership for African Social and Governance Research (PASGR) | https://www.pasgr.org/ |
| 1. CEE Joburg | https://ceejoburg.com/ |
| 1. African Union | https://au.int/ |
| 1. Alliance for Health Policy and Systems Research | http://www.who.int/alliance-hpsr/en/ |
| 1. EVIPNet | <https://www.who.int/evidence/en/> |
| 1. McMaster KT+ Database | http://plus.mcmaster.ca/kt/ |
| 1. UNICEF | <https://www.unicef-irc.org/publications> |
| 1. Human Development research foundation | https://hdrfoundation.org/evidence-synthesis-analysis-team/ |
| 1. USAID | <https://www.usaid.gov/> |
| 1. FCDO | <https://www.gov.uk/government/organisations/foreign-commonwealth-development-office> |
| 1. Oxfam | <https://www.oxfam.org/en> |
| 1. IDC | <https://idc.co.za/> |
| 1. IDRC | https://www.idrc.ca/en?gclid=Cj0KCQiA1sucBhDgARIsAFoytUuJYXCUfJGIPfRVAaw9g_CrlDY1Z-MqJWb5g0RFWhHNoX9bFvhqVnQaApwLEALw_wcB |
| 1. GIZ | <https://www.giz.de/en/html/index.html> |
| 1. Deval | <https://www.deval.org/en/> |
| 1. IRC | <https://www.rescue.org/> |
| 1. World Bank Evaluation office | <https://ieg.worldbankgroup.org/> |
| 1. FAO | <https://www.fao.org/home/en> |
| 1. OECD | <https://www.oecd.org/southafrica/> |
| 1. WHO | <https://www.who.int/> |
| 1. UNDP | <https://www.undp.org/> |
| 1. US Data coalition | <https://www.datacoalition.org/> |
| 1. US coalition for evidence-based policy | <http://coalition4evidence.org/> |
| 1. Results 4 America | <https://results4america.org/> |
| 1. Results for development | <https://r4d.org/> |
| 1. Behavioural insights team | <https://www.bi.team/> |
| 1. Alliance for Useful Evidence_NESTA | <https://www.nesta.org.uk/project/alliance-useful-evidence/> |
| 1. Agora | <https://agora.unicef.org/> |
| 1. Centre for Science and Policy | <https://www.csap.cam.ac.uk/> |
| 1. APO | \| <https://apo-opa.com/> \| \| --- \| |
| 1. Community NI | <https://www.communityni.org/> |
| 1. Epistemonikos (Chile) | [https://www.epistemonikos.org/#](https://www.epistemonikos.org/) |
| 1. Hewlett foundation | <https://hewlett.org/> |
| 1. William T Grant Foundation | <https://wtgrantfoundation.org/> |
| 1. Monash University in Australia | <https://www.monash.edu/> |
| 1. On Think Tanks | <https://onthinktanks.org/> |
| 1. Asian Development Bank | https://www.adb.org/ |
| 1. Bill & Melinda Gates Foundation | https://www.gatesfoundation.org/ |
| 1. Center for Effective Global Action Research Publications | https://vcresearch.berkeley.edu/research-unit/center-effective-global-action |
| 1. Innovations for Poverty Action Publications | https://www.poverty-action.org/publications |
| 1. Inter-American Development Bank | https://www.iadb.org/en/topics-effectiveness-improving-lives/impact-evaluations-repository |
| 1. Millennium Challenge Corporation | https://www.mcc.gov/ |
| 1. National Bureau of Economic Research, USA | https://www.nber.org |
| 1. USAID Evaluations Clearinghouse | http://dec.usaid.gov/ |
| 1. Thünen-Institute, GER | www.thuenen.de/ |
| 1. Observatory for Public Sector Innovation | https://oecd-opsi.org/bi-projects/ |
| 1. Campbell Collaboration | https://campbellcollaboration.org/ |
| 1. Data Coalition | https://www.datacoalition.org |
| 1. Bosch Stiftung | https://www.bosch-stiftung.de/de/story/es-kann-nicht-genuegend-stiftungen-geben |
| 1. Mastercard Foundation | https://mastercardfdn.org |
| 1. Evidence Commission | https://www.mcmasterforum.org/networks/evidence-commission |
| 1. COVID-END | https://www.mcmasterforum.org/networks/covid-end |
| 1. Centre for Evidence and Implementation | https://www.ceiglobal.org |
| 1. Centre for Global Development | https://www.cgdev.org |
| 1. IDInsights | https://www.idinsight.org |

**Appendix C: Descriptive data extraction tool (all included studies)**

| **Variable** | **Description** |
| --- | --- |
| Publication year | Year of study publication |
| Publication type | Publication type of study(e.g., journal articles) |
| Project name | State name of the project |
| Region (socio-economic) | World Bank country classifications by income level |
| Region (geographical) | World bank classification. |
| Country | Country(s) of intervention |
| Sector | Intervention sector   - Health - Agriculture, fishing and forestry - Social protection - Education - Industry, trade and services - Public administration - Energy and extractives - Financial sector - Water, sanitation and waste management - Transportation - Information and communications technologies |
| Nature of the evidence | Denote if the intervention focuses on the art or science of using evidence |
| Evidence Use Mechanism | EIDM interventions according to mechanisms of change namely:   - M1 Awareness: Awareness for, and positive attitudes towards EIDM - M2 Agree: Mutual understanding & agreement on evidence needs & policy-relevant evidence - M3 Access: Providing communication of, and access to, evidence - M4 Interact: Interaction between decision-makers and researchers - M5 Skills: Supporting decision-makers skills in accessing and making sense of evidence - M6 Structure & Process: Influencing decision-making structures and processes |
| Intervention description | Provide detailed description of the intervention and its different components. Include details of sections of the manuscript and page numbers where authors describe details of the intervention. |
| Intervention theory of change / logic model | Does the study mention a theory of change or logic model? If yes, provide page number and provide a short description of the theory of change and the pathway discussed by the authors which explain the reported effects. |
| Intervention implementation | Does the study describe the process of implementing the intervention or programme? If yes, please provide page number and provide a short description of the information reported in the study. |
| Stage of the policy cycle | Policy Design; Policy Implementation |
| Single vs multicomponent interventions |  |
| Year of the intervention | The earliest date (year) observations are exposed to the intervention. |
| Length of follow up | How many months have elapsed between the start of the intervention (earliest date observations are exposed to the intervention) and the date of the final outcome measurement. |
| Exposure to intervention | For how long are the observations exposed to the intervention (in months)? |
| Type of policymaker | Does the study describe the policymaking population targeted by the intervention or programme? If yes, please provide page number and provide a short description of the information reported in the study. |
| Level of policymaking | Global, Regional, National, subnational, |
| Seniority of policymaker | - junior -mid-level -senior |
| Gender of policymaker | Where reported, please comment on the gender composition of the policymakers targeted by the intervention or programme. |
| Resource / information setting | Where reported, please comment on the setting in which the policymakers targeted by the intervention or programme operate; in particular, do they have access to information (eg internet, libraries) and access to resources (eg academic databases, knowledge management systems, KT staff) |

**Appendix D: Impact evaluation critical appraisal tool (Studies included to address research question 1)**

| Methodological appraisal criteria | | | | Response | | |
| --- | --- | --- | --- | --- | --- | --- |
|  |  |  |  | Yes | No | Comment |
| *IF RANDOMISED CONTROL TRIAL, START AFTER CONFOUNDING BIAS. FOR ALL OTHER STUDY DESIGNS, START HERE.*   1. Bias in selection of participants into the study   *Are participants selected in a way that minimizes selection bias? ^^[[1]](#footnote-1)^^*  Appraisal indicators  Consider whether: | | | |  |  |  |
| 1. *there is an adequate description of how and why sample was chosen (i.e., identified/selected/recruited)* | | | |  |  |  |
| 1. *there is adequate sample size to allow for representative and/or statistically significant conclusions* | | | |  |  |  |
| 1. *participants in the control^^[[2]](#footnote-2)^^ group were sampled from the same population as that of the treatment* | | | |  |  |  |
| 1. *group allocation process minimised potential risk of bias (e.g., using computer algorithms)* | | | |  |  |  |
| 1. *the selection of participants into the study (or into the analysis) is based on participant characteristics observed after the start of the intervention* | | | |  |  |  |
| Low risk of bias | Risk of bias | High risk of bias | Critical risk of bias | *Worth to continue: Y/N?* | | |
|  | | | | | | |
| 1. Bias due to confounding   *Is confounding potentially controllable in the context of this study?*  Appraisal indicators:  Consider whether: | | | |  |  |  |
| 1. *there is potential for confounding of the effect of the intervention in this study. If yes, provide example of confounding domain in comment box.^^[[3]](#footnote-3)^^* | | | |  |  |  |
| 1. *where matching was applied, it featured sufficient criteria^^[[4]](#footnote-4)^^* | | | |  |  |  |
| 1. *where relevant, the authors conducted an appropriate analysis that controlled for all potential/remaining critical confounding domains after matching had been applied* | | | |  |  |  |
| 1. *the authors avoided adjusting for variables identified after the intervention has been administered* | | | |  |  |  |
| 1. *the treatment and control group are comparable after matching/controls have been done. Select one of the following:*   ☐No statistically significant differences  ☐Statistically significance differences  ☐Negligible descriptive differences  ☐Significant descriptive differences | | | |  |  |  |
| Low risk of bias | Risk of bias | High risk of bias | Critical risk of bias | *Worth to continue: Y/N?* | | |
|  | | | | | | |
| *IF RANDOMISED CONTROL TRIAL, SKIP I + II (ABOVE) AND START HERE!*  Bias due to confounding (because of ineffective randomisation)  *Is allocation of treatment status truly random?*  Appraisal indicators  Consider whether: | | | |  |  |  |
| 1. *eligibility criteria for study entry are specified* | | | |  |  |  |
| 1. *there is a clear description of the randomisation process and methods are robust* | | | |  |  |  |
| 1. *the unit of randomisation and number of participants is clearly stated (pay special attention to treatment and control locations/ balance)* | | | |  |  |  |
| 1. *characteristics of both baseline and endline sample are provided^^[[5]](#footnote-5)^^and at endline the treatment and control group are comparable. Select one of the following:*   ☐No statistically significant differences  ☐Statistically significance differences  ☐Negligible descriptive differences  ☐Significant descriptive differences | | | |  |  |  |
| Low risk of bias | Risk of bias | High risk of bias | Critical risk of bias | *If critical risk of bias, treat as non-random study* | | |
|  | | | | | | |
| 1. Bias due to departures from intended interventions   *Was the intervention implemented as laid out in the study protocol?*  Appraisal indicators  Consider whether: | | | |  |  |  |
| 1. *the critical co-interventions were balanced across intervention and control groups* | | | |  |  |  |
| 1. *treatment switches were low enough to not threaten the validity of the estimated effect of the intervention* | | | |  |  |  |
| 1. *implementation failure was minor and unlikely to threaten the validity of the estimated effect of the intervention* | | | |  |  |  |
| 1. *it is possible that the intervention was taken by the controls (contamination and possible crossing-over)^^[[6]](#footnote-6)^^* | | | |  |  |  |
| 1. *it is possible that knowledge of group allocation affects how the two study groups are treated during delivery and evaluation of the intervention^^[[7]](#footnote-7)^^* | | | |  |  |  |
| Low risk of bias | Risk of bias | High risk of bias | Critical risk of bias | *Worth to continue: Y/N?* | | |
|  | | | | | | |
| 1. Bias due to missing/incomplete data (attrition)   *Are the intervention and control groups free of critical differences in participants with missing/incomplete data?*  Appraisal indicators  Consider whether: | | | |  |  |  |
| 1. *outcome data are reasonably complete (80% or above)^^[[8]](#footnote-8)^^* | | | |  |  |  |
| 1. *If level of attrition (or other forms of missing/incomplete data) is more than 20%, are reasons for the missing data reported?* | | | |  |  |  |
| 1. *If level of attrition (or other forms of missing/incomplete data) is more than 20%, do the authors demonstrate similarity between remaining participants and those lost to attrition and are the proportion of participants with missing/incomplete data and reasons for missing/incomplete data similar across groups?* | | | |  |  |  |
| 1. *If level of attrition (or other forms of missing/incomplete data) is more than 20%, were appropriate statistical methods used to account for missing data? (e.g., sensitivity analysis)^^[[9]](#footnote-9)^^* | | | |  |  |  |
| 1. *If not possible to control for missing/incomplete data, are outcomes with missing/incomplete data excluded from analysis?* | | | |  |  |  |
| Low risk of bias | Risk of bias | High risk of bias | Critical risk of bias | *Worth to continue: Y/N?* | | |
|  | | | | | | |
| 1. Bias in measurement of outcomes   *Are measurements appropriate, e.g., clear origin, or validity known?*  Appraisal indicators  Consider whether: | | | |  |  |  |
| 1. *there was an adequate period for follow up^^[[10]](#footnote-10)^^* | | | |  |  |  |
| 1. *the outcome measure (e.g., employment status, income) was clearly defined and objective^^[[11]](#footnote-11)^^* | | | |  |  |  |
| 1. *outcomes were assessed using standardised instruments and indicators* | | | |  |  |  |
| 1. *outcome measurements reflect what the experiment set out to measure* | | | |  |  |  |
| 1. *the methods of outcome assessment were comparable across groups* | | | |  |  |  |
| 1. *were outcome assessors aware of the intervention received by study participants?^^[[12]](#footnote-12)^^* | | | |  |  |  |
| Low risk of bias | Risk of bias | High risk of bias | Critical risk of bias | *Worth to continue: Y/N?* | | |
|  | | | | | | |
| 1. Bias in selection of results reported   *Are the reported outcomes consistent with the proposed outcomes at the protocol stage?*  Appraisal indicators  Consider whether: | | | |  |  |  |
| 1. *it is unlikely that the reported effect estimate has been selected for publication due to it being a particularly notable finding among numerous exploratory analyses* | | | |  |  |  |
| 1. *it is unlikely that the reported effect estimate is prone to selective reporting from among multiple outcome measurements within the outcome domain* | | | |  |  |  |
| 1. *it is unlikely that the reported effect estimate is prone to selective reporting from among multiple analyses of the outcome measurements, including sub-group analysis* | | | |  |  |  |
| 1. *if sub-group/ancillary/adjusted analyses are presented, are these pre-specified or exploratory?* | | | |  |  |  |
| 1. *the analysis includes an intention to treat analysis. (If so, was this appropriate and were appropriate methods used to account for missing data?)^^[[13]](#footnote-13)^^* | | | |  |  |  |
| 1. *do the authors report on all variables they aimed to study (as specified in their protocol or study aims/research questions)?* | | | |  |  |  |
| Low risk of bias | Risk of bias | High risk of bias | Critical risk of bias |  | | |
| OVERALL RISK OF BIAS: | | | | | | |
| *Assigning of overall risk of bias score:*   - *A single critical rating in either domain automatically leads to a critical risk of bias score.* - *A threshold of two ratings of the same level automatically leads to an upgrading of the overall risk of bias. E.g., if a study has two moderate rankings the overall risk of bias is at least moderate; if the study has had two high rankings, it is rated at an overall high risk of bias.* | | | | | | |

**Appendix E: Qualitative and mixed-method critical appraisal tool (Studies included to address research question 2)**

| **Study type** | **Methodological appraisal criteria** | | | | | | | | | | | | | | | **Response** | | | | | | |
| --- | --- | --- | --- | --- | --- | --- | --- | --- | --- | --- | --- | --- | --- | --- | --- | --- | --- | --- | --- | --- | --- | --- |
|  |  |  |  |  |  |  |  |  |  |  |  |  |  |  |  | Yes | No | Comment | | | | |
| *Screening questions: assessing ‘fatal flaws’ (Dixon-Woods 2005)*  *Configurative ‘fatal flaws’ based on Pawson (2003) TAPUS framework* | Configurative assessment:   - Study reports primary data and applied methods - Study states clear research questions and objectives - Study states clear research design, which is appropriate to address the stated research question and objectives (*Purposivity*) - The findings of the study are based on collected data, which justify the knowledge claims (*Accuracy*) | | | | | | | | | | | | | | |  |  |  | | | | |
|  | ***Screening question based on abstract and/or superficial reading of full-text: Further appraisal is not feasible or appropriate when the answer is ‘No’ to any of the above screening questions!*** | | | | | | | | | | | | | | | | | | | | | |
|  | | | | | | | | | | | | | | | | | | | | | | |
| **Study type** | **Methodological appraisal criteria** | | | | | | | | | | | | | | | **Response** | | | | | | |
|  |  |  |  |  |  |  |  |  |  |  |  |  |  |  |  | Yes | No | Comment / Confidence judgment | | | | |
| *1. Qualitative and descriptive quantitative, and process evaluations* | 1. **RESEARCH IS DEFENSIBLE IN DESIGN** (providing a research strategy that addresses the question)   Appraisal indicators:   - *Is the research design clearly specified and appropriate for aims and objectives of the research?*   Consider whether | | | | | | | | | | | | | | |  |  |  | | | | |
|  | 1. *there is a discussion of the rationale for the study design* | | | | | | | | | | | | | | |  |  |  | | | | |
|  | 1. *the research question is clear, and suited to the inquiry* | | | | | | | | | | | | | | |  |  |  | | | | |
|  | 1. *there are convincing arguments for different features of the study design* | | | | | | | | | | | | | | |  |  |  | | | | |
|  | 1. *limitations of the research design and implications for the research evidence are discussed* | | | | | | | | | | | | | | |  |  |  | | | | |
|  | **Defensible** | | | **Arguable** | | | **Critical** | | | | | | | | **Not defensible** | *Worth to continue:* | | | | | | |
|  |  | | | | | | | | | | | | | | | | | | | | | |
|  | 1. **RESEARCH FEATURES AN APPROPRIATE SAMPLE** (following an adequate strategy for selection of participants)   Appraisal indicators:  Consider whether | | | | | | | | | | | | | | |  |  |  | | | | |
|  | 1. *there is a description of study location and how/why it was chosen* | | | | | | | | | | | | | | |  |  |  | | | | |
|  | 1. *the researcher has explained how the participants were selected* | | | | | | | | | | | | | | |  |  |  | | | | |
|  | 1. *the selected participants were appropriate to collect rich and relevant data* | | | | | | | | | | | | | | |  |  |  | | | | |
|  | 1. *reasons are given why potential participants chose not take part in study* | | | | | | | | | | | | | | |  |  |  | | | | |
|  | **Appropriate sample** | | | | **Functional sample** | | | | **Critical sample** | | | | | **Flawed sample** | | *Worth to continue:* | | | | | | |
|  |  | | | | | | | | | | | | | | | | | | | | | |
|  | 1. **RESEARCH IS RIGOROUS IN CONDUCT**   (providing a systematic and transparent account of the research process)  Appraisal indicators:  Consider whether | | | | | | | | | | | | | | |  |  |  | | | | |
|  | 1. *researchers provide a clear account/description of the process by which data was collected (e.g. for interview method, is there an indication of how interviews were conducted?/procedures for collection or recording of data?)* | | | | | | | | | | | | | | |  |  |  | | | | |
|  | 1. *researchers demonstrate that data collection targeted depth, detail and richness of information (e.g. interview/observation schedule)* | | | | | | | | | | | | | | |  |  |  | | | | |
|  | 1. *there is evidence of how descriptive analytical categories, classes, labels, etc. have been generated and used* | | | | | | | | | | | | | | |  |  |  | | | | |
|  | 1. *presentation of data distinguishes clearly between the data, the analytical frame used, and the interpretation* | | | | | | | | | | | | | | |  |  |  | | | | |
|  | 1. *methods were modified during the study; and if so, has the researcher explained how and why?* | | | | | | | | | | | | | | |  |  |  | | | | |
|  | **Rigorous conduct** | | | **Considerate conduct** | | | | | | **Critical conduct** | | **Flawed conduct** | | | | *Worth to continue:* | | | | | | |
|  |  | | | | | | | | | | | | | | | | | | | | | |
|  | 1. **RESEARCH FINDINGS ARE CREDIBLE IN CLAIM/BASED ON DATA**   (providing well-founded and plausible arguments based on the evidence generated)  Appraisal indicators:  Consider whether | | | | | | | | | | | | | | |  |  |  | | | | |
|  | 1. *there is a clear description of the form of the original data* | | | | | | | | | | | | | | |  |  |  | | | | |
|  | 1. *sufficient amount of data are presented to support interpretations and findings/conclusions* | | | | | | | | | | | | | | |  |  |  | | | | |
|  | 1. *the researchers explain how the data presented were selected from the original sample to feed into the analysis process (i.e. commentary and cited data relate; there is an analytical context to cited data, not simply repeated description; is there an account of frequency of presented data?)* | | | | | | | | | | | | | | |  |  |  | | | | |
|  | 1. *there is a clear and transparent link between data, interpretation, and findings/conclusion* | | | | | | | | | | | | | | |  |  |  | | | | |
|  | 1. *there is evidence (of attempts) to give attention to negative cases/outliers etc.* | | | | | | | | | | | | | | |  |  |  | | | | |
|  | **Credible claims** | | | **Arguable claims** | | | | **Doubtful claims** | | | | **Not credible** | | | | *If findings not credible, can data still be used?* | | | | | | |
|  |  | | | | | | | | | | | | | | | | | | | | | |
|  | 1. **REASEARCH ATTENDS TO CONTEXTS**   (describing the contexts and particulars of the study)  Appraisal indicators:  Consider whether | | | | | | | | | | | | | | |  |  |  | | | | |
|  | 1. *there is an adequate description of the contexts of data sources and how they are retained and portrayed?* | | | | | | | | | | | | | | |  |  |  | | | | |
|  | 1. *participants’ perspectives/observations are placed in personal contexts* | | | | | | | | | | | | | | |  |  |  | | | | |
|  | 1. *appropriate consideration is given to how findings relate to the contexts (how findings are influenced by or influence the context)* | | | | | | | | | | | | | | |  |  |  | | | | |
|  | 1. *the study makes any claims (implicit or explicit) that infer generalisation (if yes, comment on appropriateness)* | | | | | | | | | | | | | | |  |  |  | | | | |
|  | **Context central** | | | **Context considered** | | | | | **Context mentioned** | | | | **No context attention** | | |  | | | | | | |
|  |  | | | | | | | | | | | | | | | | | | | | | |
|  | 1. **RESEARCH IS REFLECTIVE**   (assessing what factors might have shaped the form and output of research)  Appraisal indicators:  Consider whether | | | | | | | | | | | | | | |  |  |  | | | | |
|  | 1. *appropriate consideration is given to how findings relate to researchers’ influence/own role during analysis and selection of data for presentation* | | | | | | | | | | | | | | |  |  |  | | | | |
|  | 1. *researchers have attempted to validate the credibility of findings (e.g. triangulation, respondent validation, more than one analyst)* | | | | | | | | | | | | | | |  |  |  | | | | |
|  | 1. *researchers explain their reaction to critical events that occurred during the study* | | | | | | | | | | | | | | |  |  |  | | | | |
|  | 1. *researchers discuss ideological perspectives/values/philosophies and their impact on the methodological or other substantive content of the research (implicit/explicit)* | | | | | | | | | | | | | | |  |  |  | | | | |
|  | **Reflection** | | **Consideration** | | | | **Acknowledgement** | | | | | **Unreflective research** | | | | *NB: Can override previous exclusion!* | | | | | | |
| **OVERALL CRITICAL APPRAISAL DECISION**  **Decision rule:**  - a single critical appraisal judgement^^[[14]](#footnote-14)^^ in any of the 6 appraisal domains leads to a critical overall judgement.  - 2 or more high critical appraisal judgements in any of the 6 appraisal domains lead to an overall high risk of bias / low quality rating.  - 2 or more moderate critical appraisal judgements in any of the 6 appraisal domains lead to an overall moderate risk of bias / moderate quality rating.  - which means that for a study to be rated of low risk of bias / high quality at least 5 appraisal domains need be rated as of low critical appraisal. | | | | | | | | | | | | | | | | | | | | | | |
| **HIGH QUALITY**  **EMPIRICAL RESEARCH**  (study generates new evidence relevant to the review question and complies with all methodological criteria to ensure reliability and empirical grounding of the evidence). | | **MODERATE QUALITY**  **EMPIRICAL RESEARCH**  (study generates new evidence relevant to the review question and complies with reasonable methodological criteria to ensure reliability and empirical grounding of the evidence). | | | | | | | | | **LOW QUALITY**  **EMPIRICAL RESEARCH**  (study generates new evidence relevant to the review question and complies with minimum methodological criteria to ensure reliability and empirical grounding of the evidence). | | | | | | | | **CRITICAL QUALITY**  **EMPIRICAL RESEARCH**  (the evidence generated by the study does not comply with minimum methodological criteria to ensure reliability and empirical grounding of the evidence). | | | |
|  | | | | | | | | | | | | | | | | | | | | | | |
| Sources used in this section (in alphabetical order); Campbell et al (2003); CASP (2006); CRD (2009); Dixon-Woods et al (2005); Dixon-Woods et al (2006) ; Greenhalgh & Brown (2014); Harden et al (2004); Harden et al (2009); Harden & Gough (2012); Mays & Pope (1995); Pluye et al (2011); Spencer et al 2006; Thomas et al (2003); SCIE (2010). | | | | | | | | | | | | | | | | | | | | | | |
|  | | | | | | | | | | | | | | | | | | | | | | |
|  | | | | | | | | | | | | | | | | | | | | | | |
| **Study type** | | | | | | **Methodological appraisal criteria** | | | | | | | | | | | | | | **Response** | | |
|  |  |  |  |  |  |  |  |  |  |  |  |  |  |  |  |  |  |  |  | Yes | No | Comment /confidence judgment |
| *2. Mixed-methods^2^*  *Sequential explanatory design*  *The quantitative component is followed by the qualitative. The purpose is to explain quantitative results using qualitative findings. E.g., the quantitative results guide the selection of qualitative data sources and data collection, and the qualitative findings contribute to the interpretation of quantitative results.*  *Sequential exploratory design The qualitative component is followed by the quantitative. The purpose is to explore, develop and test an instrument (or taxonomy), or a conceptual framework (or theoretical model). E.g., the qualitative findings inform the quantitative data collection, and the quantitative results allow a generalization of the qualitative findings.*  *Triangulation designs The qualitative and quantitative components are concomitant. The purpose is to examine the same phenomenon by interpreting qualitative and quantitative results (bringing data analysis together at the interpretation stage), or by integrating qualitative and quantitative datasets (e.g., data on same cases), or by transforming data (e.g., quantization of qualitative data).*  *Embedded/convergent design The qualitative and quantitative components are concomitant. The purpose is to support a qualitative study with a quantitative sub-study (measures), or to better understand a specific issue of a quantitative study using a qualitative sub-study, e.g., the efficacy or the implementation of an intervention based on the views of participants.* | | | | | | 1. **RESEARCH INTEGRATION/SYNTHESIS OF METHODS**   (assessing the value-added of the mixed-methods approach)  Applied mixed-methods design:   - Sequential explanatory design - Sequential explorative design - Triangulation design - Embedded design   Appraisal indicators:  Consider whether | | | | | | | | | | | | | |  |  |  |
|  |  |  |  |  |  | 1. *the rationale for integrating qualitative and quantitative methods to answer the research question is explained*   *[DEFENSIBLE]* | | | | | | | | | | | | | |  |  |  |
|  |  |  |  |  |  | 1. *the mixed-methods research design is relevant to address the qualitative and quantitative research questions, or the qualitative and quantitative aspects of the mixed methods research question*   *[DEFENSIBLE]* | | | | | | | | | | | | | |  |  |  |
|  |  |  |  |  |  | 1. *there is evidence that data gathered by both research methods was brought together to inform new findings to answer the mixed-methods research question (e.g. form a complete picture, synthesise findings, configuration)*   *[CREDIBLE]* | | | | | | | | | | | | | |  |  |  |
|  |  |  |  |  |  | 1. *the approach to data integration is transparent and rigorous in considering all findings from both the qualitative and quantitative module (danger of cherry-picking)*   *[RIGOROUS]* | | | | | | | | | | | | | |  |  |  |
|  |  |  |  |  |  | 1. *appropriate consideration is given to the limitations associated with this integration, e.g., the divergence of qualitative and quantitative data (or results)?*   *[REFLEXIVE]* | | | | | | | | | | | | | |  |  |  |
| For mixed-methods research studies, each component undergoes its individual critical appraisal first. Since qualitative studies are either included or excluded, no combined risk of bias assessment is facilitated, and the assigned risk of bias from the quantitative component similarly holds for the mixed-methods research.  The above appraisal indicators only refer to the applied mixed-methods design. If this design is not found to comply with each of the four mixed-methods appraisal criteria below, then the quantitative/qualitative components will individually be included in the review: | | | | | | | | | | | | | | | | | | | | | | |
| Mixed-methods critical appraisal:   1. Research is defensible in design 2. Research is rigorous in conduct 3. Research is credible in claim 4. Research is reflective | | | | | | Qualitative critical appraisal:  Include / Exclude | | | | | | | | | | Quantitative critical appraisal:   1. Low risk of bias 2. Risk of bias 3. High risk of bias 4. Critical risk of bias | | | | | | |
| Combined appraisal:  Include / Exclude mixed-methods findings judged with ____________________________ risk of bias | | | | | | | | | | | | | | | | | | | | | | |
|  | | | | | | | | | | | | | | | | | | | | | | |
| Section based on Pluye et al (2011). Further sources consulted (in alphabetical order): Creswell & Clark (2007); Crow (2013); Long (2005); O’Cathain et al (2008); O’Cathain (2010); Pluye & Hong (2014); Sirriyeh et al (2011). | | | | | | | | | | | | | | | | | | | | | | |

**Appendix F: Effect size data extraction tool (Studies included to address research question 1)**

| Method information | Evaluation Design | Select one of the options below:   1. Experimental (defined as prospective randomised assignment, where randomisation is implemented by researchers (or by decision makers in the context of an evaluation study)  2. Quasi-experimental (including natural experiments and non-randomised studies) |
| --- | --- | --- |
|  | Evaluation Method | ● If Experimental, then select: i) Randomised controlled trial  ● If Quasi-experiment or natural experiment, then select one of the following: i) Natural experiment in which exposure to treatment is random; ii) Regression Discontinuity Design (RDD); iii) Difference-in-Differences (DID) / Fixed effects estimation; iv) Instrumental variable (IV) estimation; v) Endogenous treatment-effects models (including endogenous switching regression, and other methods synonymous to the Heckman two step model); vi) Statistical matching (includes PSM or statistical weighting) vii) Interrupted time series (ITS); viii) Synthetic controls |
|  | Method description | Provide a brief description of the method applied and note if any methods have been combined. |
|  | Study population | Provide any details in the paper that describe how the study population was selected, covering:  a) How is the population selected? what is the sampling strategy to recruit participants from that population into the study?  b) What are the characteristics of study participants? |
|  | Additional methods | Describe any additional methods used in analysis. If none, select not applicable. |
| Estimate information | Analysis type for this effect size | Free text, what type of analysis was used (OLS regression, Probit regression, 2SLS, ANCOVA, etc.) |
|  | Treatment effect estimated | 1=Intention to Treat (ITT), 2=Average Treatment Effect on the Treated (ATET), 3=Average Treatment Effect (ATE) 4 = Local Average Treatment Effect (LATE), 5 =Other |
|  | Treatment effect estimated other | Provide details if other treatment effect estimated  Also include the relevant matching algorithm here (ex: kernel, nearest neighbour, etc...) |
|  | Unit of analysis | What is the unit of analysis? UOA for this effect size: 1= Individual, 2 = Department (e.g. department within a governmen ministry), 3 = Geographical area, 4 = Other, 5 = Not clear  If OTHER, ALWAYS PLEASE SPECIFY with a comment in this cell. |
|  | Covariate adjustment | Did the regression specification control for variables other than the treatment variable?: 1= Yes; 0 = No. This includes matching variables. |
|  | Covariate adjustment description | List the control variables included in the specific specification related to the estimate (including any noted fixed effects). |
|  | Source | Note the page number, table number, column, and row you used to extract the estimate data [Open Answer] |
| Outcome dataset | Outcome code | Choose an outcome code for each corresponding effect size: i) Evidence use ii) Socio-economic impact iii) Intermediate |
|  | Outcome sub-group | Choose an outcome sub-group code for each corresponding estimate:  ● Evidence use: 1. Evidence use for policy design 2. Evidence use for policy implementation ● Socio-economic impact ● Intermediate: 1.Capability to use evidence; 2. Motivation to use evidence; 3. Opportunity to use evidence |
|  | Outcome description | Record the outcome for the corresponding effect size. Use this open answer field to enter, in the author’s own words, a description of the outcome. Be selective and concise with the excerpts being transcribed here as to ensure accurate and precise descriptions of the outcome. Include information about the unit of the outcome and how it has been measured. Include page numbers with every excerpt extracted. |
|  | Outcome measurement | How was the data collected? 1=Self-reported, 2=Administrative data |
|  | Outcome type | Record the type of outcome variable: 1=Continuous; 2=Discrete (including proportions); 3=Nominal (binary); 4=Ordinal (binary); 5=Nominal (non-binary); 6=Ordinal (non-binary); 7=Interval. |
|  | Levels or changes | 0 = Unit is the level of outcome variable, 1 = Change in outcome variable |
|  | Reverse sign | Record no=0 if an increase is good, record yes=1 if a decrease is good and the sign needs to be reversed (i.e., decrease is good) |
|  | Outcome dataset | Record if data for this outcome comes from an identified dataset |
| Treatment variable information | Treatment | Record the treatment variable as written in the model (e.g., the variable name the author uses). This column enables to distinguish what treatment is evaluated here for this specific estimate. This is very important as many studies have multiple treatments. |
|  | Treatment type | Describe the types of treatment variable used: i) binary; ii) continuous; iii) categorical; iv) other |
|  | Comparison | 1=No intervention (service delivery as usual), 2=Other intervention, 3=Pipeline (waitlist) control (still service delivery as usual) 4. Other |
|  | Describe comparison group | Describe the comparison group |
|  | Subgroup | Is this analysis of a subgroup or estimating heterogeneous effects?  0=no, 1=yes |
|  | Subgroup information | Describe the subgroup or variable interacted with the treatment variable. If no subgroup or heterogeneity analysis, select not applicable |
| Estimate data | Mean treatment (Pre) | Outcome mean for the treatment group (pre-intervention) |
|  | SD treatment (Pre) | Outcome standard deviation for treatment group (pre-intervention) |
|  | Mean Control (Pre) | Outcome mean for the comparison group (pre-intervention) |
|  | SD Control (Pre) | Outcome standard deviation for control group (pre-intervention) |
|  | Mean treatment (Post) | Outcome mean for the treatment group (post-intervention) |
|  | SD treatment (Post) | Outcome standard deviation for treatment group (post-intervention) |
|  | Mean Control (Post) | Outcome mean for the comparison group (post-intervention) |
|  | SD Control (Post) | Outcome standard deviation for control group (post-intervention) |
|  | SD pooled (Pre) | Outcome standard deviation for pooled group (treatment and control) (pre-intervention) |
|  | SD pooled (Post) | Outcome standard deviation for pooled group (treatment and control) (post-intervention) |
|  | SD pooled (PP) | Outcome standard deviation for pooled group (treatment and control) (includes pre and post intervention data) |
|  | Mean difference | Overall mean difference (treatment - control) |
|  | SE difference | Standard error of the overall mean difference |
|  | Tstat difference | t-statistic of mean difference |
|  | Odds ratio | Odds ratio reported in the study |
|  | SE odds ratio | Odds ratio standard error reported in the study |
|  | Risk ratio | Risk ratio reported in study |
|  | SE Risk Ratio | Risk ratio standard error |
|  | Coeff reg | Report the regression coefficient of the treatment effect |
|  | SE reg | Report the associated standard error of the regression coefficient. |
|  | Tstat reg | Report the associated t statistic of the effect size (coefficient/SE) |
|  | CI_LB reg | Report the associated Lower bound of the 95% Confidence interval of the effect size. If CI is reported for a different confidence level, indicate that in the notes section. |
|  | CI_UP reg | Report the associated Upper bound of the 95% Confidence interval of the effect size. If CI is reported for a different confidence level, indicate that in the notes section. |
|  | P value exact | Exact p value if given, if not, record as written in the manuscript (e.g., p < .001, or p > .05) |
|  | Interaction term 1 coeff | Required if requested to extract information from an interaction term (in addition to a single term) |
|  | Interaction term 1 SE | Required if requested to extract information from an interaction term (in addition to a single term) |
|  | Interaction term 1 Tstat | Required if requested to extract information from an interaction term (in addition to a single term) |
|  | Interaction term 1 CI_LB | Required if requested to extract information from an interaction term (in addition to a single term) |
|  | Interaction term 1 CI_UP | Required if requested to extract information from an interaction term (in addition to a single term) |
|  | Interaction term 1 P value exact | Required if requested to extract information from an interaction term (in addition to a single term) |
|  | Clusters treatment | Number of clusters - treatment group |
|  | Clusters control | Number of clusters -  control group |
|  | Clusters total | Number of clusters - total sample |
|  | N treatment | Sample size - treatment group |
|  | N control | Sample size - control group |
|  | N total | Sample size - total sample |
|  | periods | Record how many time points (e.g.measurement points) there are in the analysis (e.g., cross sectional data is 1, panel data with 3 measurements is 3). |
|  | Does the sample size need to be corrected? | Often in panel data, models will report number of observations rather than number of participants. In this column you will indicate 1="Yes" if the sample size needs to be divided by the number of periods, and 0="No" if either it is cross-sectional data, or if the authors have already divided the number of observations by the number of panel assessments and thus no correction is necessary. |
|  | Source | Note the page number, table number, column, and row you used to extract the data |
| Other | Other linked to previous columns | Provide any other relevant information from the study |
| Qualitative data | Hypothesised mechanism of action | Does the study mention a theory of change? If yes, provide page number and provide a short description of the theory of change and the mechanisms discussed by the authors which explain the reported effects. |
|  | Unintended consequences | Describe any unintended consequences. These could be quantified or observed. They can include unexpected patterns in outputs / outcomes. Equity considerations can be listed here (ex. women were unintentionally excluded from the intervention). |
|  | Outcomes | Present any relevant outcomes that have not been captured in the Outcome description column of the Quant tool. This could include outcomes analysed using methods excluded based on our study design criteria. |
|  | Sources of bias and limitations | Report any author reported bias or limitations of the study |

1. Selection bias can occur both in the way that individuals are accepted for participation in a study, and in the way that ‘treatment’ is assigned to individuals once they have been accepted into a study. This section deals with both these understandings of selection bias. [↑](#footnote-ref-1)
2. The terms ‘control’ and ‘comparison’ group refer to any group with which the treatment of interest is compared that is presumed to represent conditions in the absence of that treatment, whether a true random control or not. [↑](#footnote-ref-2)
3. Confounding domains are those for which, in the context of this study, adjustment is expected to lead to an important change in the estimated effect of the intervention. [↑](#footnote-ref-3)
4. Matching can be done on the calculated propensity score or covariates. If the latter, it should ideally be done on pre-test measures and other characteristics, such as demographic. Answer ‘no’ if the study only matched on pre-test measures of some or all variables used later as outcome measures OR matched only on endline characteristics*.*  [↑](#footnote-ref-4)
5. Preferable condition: A RCT with appropriate randomisation procedure can be included without showing baseline data, as both experimental groups can be assumed to be equal at baseline by design. [↑](#footnote-ref-5)
6. Whilst challenging in terms of estimating impact, spill-overs might be an important finding in itself. [↑](#footnote-ref-6)
7. Consider only in extreme cases in which preferential treatment is clearly evident; blinding is generally not expected in social interventions. [↑](#footnote-ref-7)
8. The assumption here that the level of attrition (or other forms of missing/incomplete data) is sufficiently low to not require adjustment. [↑](#footnote-ref-8)
9. Select ‘no’ if the study addresses missing/incomplete data through simple estimates of missing data and observations. [↑](#footnote-ref-9)
10. In many social science interventions, follow-up is not required to coincide with the start of the treatment; further, longer periods of follow up are often required to measure changes. [↑](#footnote-ref-10)
11. Subjective measures (e.g. those based on self-report) are likely to have lower reliability and validity than objective measures. [↑](#footnote-ref-11)
12. Consider only in extreme cases in which preferential treatment is clearly evident; blinding is generally not expected in social interventions. [↑](#footnote-ref-12)
13. Usually in clinical RCTs, rare in social science: only rate if conducted. [↑](#footnote-ref-13)
14. For the qualitative studies, we use a slightly different language to scale the critical appraisal assessments as compared to the quantitative studies. The far right rating column always reflects a ‘critical’ appraisal judgement (i.e. ‘unreflective research’ above) with judgements moving further to the left on a scale from high to low critical appraisal. [↑](#footnote-ref-14)
